# Hsp90 Activity Is Necessary for the Maturation of Rabies Virus Polymerase

**DOI:** 10.3390/ijms23136946

**Published:** 2022-06-22

**Authors:** Iga Dalidowska, Anna Orlowska, Marcin Smreczak, Pawel Bieganowski

**Affiliations:** 1 Mossakowski Medical Research Institute, Polish Academy of Sciences, 02-106 Warsaw, Poland; idalidowska@imdik.pan.pl; 2 Department of Virology, National Veterinary Research Institute, 24-100 Puławy, Poland; anna.orlowska@piwet.pulawy.pl (A.O.); smreczak@piwet.pulawy.pl (M.S.)

**Keywords:** Hsp90, heat shock protein 90, rabies virus, large protein, phosphoprotein

## Abstract

*Mononegavirales* is an order of viruses with a genome in the form of a non-segmented negative-strand RNA that encodes several proteins. The functional polymerase complex of these viruses is composed of two proteins: a large protein (L) and a phosphoprotein (P). The replication of viruses from this order depends on Hsp90 chaperone activity. Previous studies have demonstrated that Hsp90 inhibition results in the degradation of mononegaviruses L protein, with exception of the rabies virus, for which the degradation of P protein was observed. Here, we demonstrated that Hsp90 inhibition does not affect the expression of rabies L and P proteins, but it inhibits binding of the P protein and L protein into functional viral polymerase. Rabies and the vesicular stomatitis virus, but not the measles virus, L proteins can be expressed independently of the presence of a P protein and in the presence of an Hsp90 inhibitor. Our results suggest that the interaction of L proteins with P proteins and Hsp90 in the process of polymerase maturation may be a process specific to particular viruses.

## 1. Introduction

The production of large quantities of the proteins during replication of a virus requires help from cellular chaperones, such as Hsp90. Hsp90 chaperone activity is necessary for the efficient replication of all viruses tested so far, but the proteins that interact with Hsp90 seem to be virus-specific [1,2]. The universal dependence of viruses on Hsp90 makes this protein an attractive target for pharmacological intervention in viral infections. Hsp90 activity depends on the ATP hydrolysis catalyzed by the ATPase located in its N-terminal domain [3,4]. One of the first ATPase inhibitors of Hsp90 successfully used to inhibit Hsp90 chaperone activity in cultured mammalian cells was 17-N-allylamino-17-demethoxygeldanamycin (17-AAG) [5]. Many Hsp90-specific inhibitors have been discovered in recent years and some of them were, or are currently, tested in clinical trials, making a perspective of antiviral use of these compounds even more appealing [6,7].

*Mononegavirales* is an order of enveloped viruses that have a genome in the form of a nonsegmented negative-strand RNA. Many important pathogens belong to this order, such as the Ebola virus, measles virus (MeV), mumps virus (MuV), and lyssaviruses, with the prototype rabies virus (RABV) causing rabies that, nowadays, is often neglected, particularly in highly developed countries. However, more than 60,000 human deaths each year are estimated by the WHO, despite the availability of both pre- and post-exposure tools [8]. Clinical infection with the rabies virus is still fatal and invariable despite the multiple in vitro and in vivo studies on the treatment of RABV infection [9,10,11]. Targeted research using a broad spectrum of antivirals, as well as the use of combination therapies, including blocking viral replication and detrimental host immune response, requires a detailed understanding of the mechanisms of RABV replication and the pathogenesis of RABV infections. There is still a need for research to further the knowledge of rabies in order to look for its effective treatment in clinical phase of the disease.

The genome of mononegaviruses encodes several proteins, including nucleoprotein (N), phosphoprotein (P), matrix protein (M), glycoprotein (G), and large protein (L). The RNA-dependent RNA polymerase of mononegaviruses in its active form is a complex composed of L and P proteins. L protein is a catalytic subunit that has domains required for RNA synthesis, mRNA capping, and polyadenylation, whereas P protein is a non-enzymatic cofactor necessary for L protein activity [12].

Studies on the Hsp90 involvement in mononegaviruses replication concluded that the activity of this chaperone is required for the proper folding and stability of the L protein, and the expression of L protein also required the presence of P protein [13,14,15].

A study of the influence of Hsp90 inhibition on RABV replication indicated that the P protein of this virus and other lyssaviruses was the main subject of Hsp90’s chaperone activity [16]. These results indicated that contrary to the L proteins of other mononegaviruses, RABV L protein was not chaperoned by Hsp90 directly, but Hsp90 was necessary for L expression because of P Hsp90-dependence. The lyssavirus polymerase complex might be an unexpected exception, considering the relatively well-conserved structure of this protein among mononegaviruses. Therefore, we decided to investigate if the L protein of the rabies virus depends on Hsp90 chaperone activity.

## 2. Results

### 2.1. RABV Infection Does Not Result in the Increased Expression of Hsp90 and Cdc37

**Figure 1:**
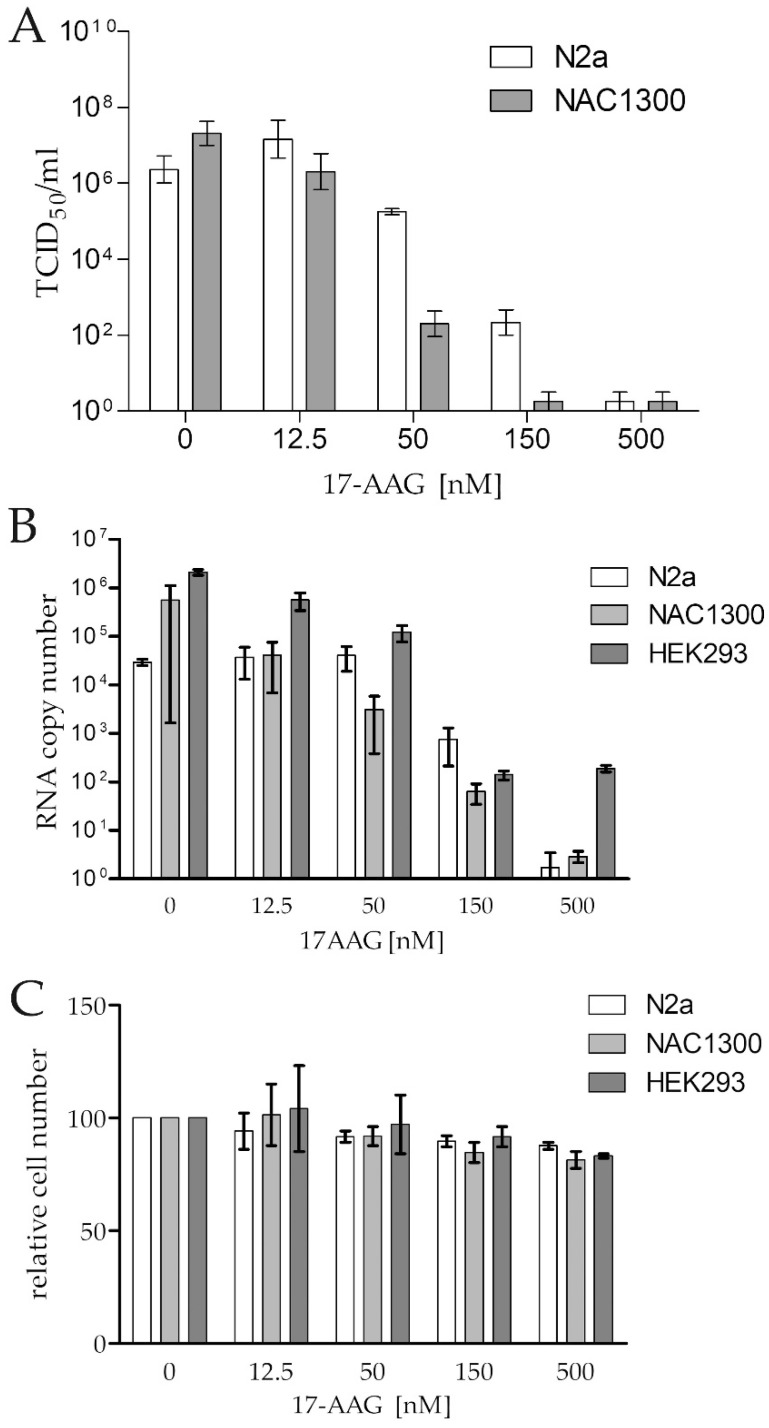
Antiviral activity of 17-AAG against RABV (MOI = 0.1) in N2a, NAC1300, and HEK293 cell monolayers demonstrated by (**A**) a reduction of RABV titre, expressed as TCID_50_/mL, and (**B**) a reduction of N gene copy numbers in the infected cell supernatant 48 h after infection. (**C**) The relative number of the uninfected cells cultured for 48 h in the presence of 17-AAG compared to the control cultures without 17-AAG. The means and ranges of the experiment (performed in duplicate) are plotted.

The strong induction of Hsp90 and Cdc37 expression caused by RABV infection has been reported [16]. We attempted to test this finding. First, we demonstrated that the CVS-11 strain effectively infected N2a, NAC1300, and HEK293 cells, and that the infection was inhibited by 17-AAG in a dose-dependent manner (Figure 1A,B). The virus RNA copy number was reduced in all cell lines by about 10,000-fold with 500 nM 17-AAG, without a significant drug-induced loss of viability of the cells (Figure 1B,C). Next, we tested the expression of Hsp90 and Cdc37 in the infected and not infected cells, and we found no indication of the increased expression of these proteins in the infected cells (Figure S1).

### 2.2. None of the RABV Proteins Are Destabilized by Hsp90 Inhibition

**Figure 2:**
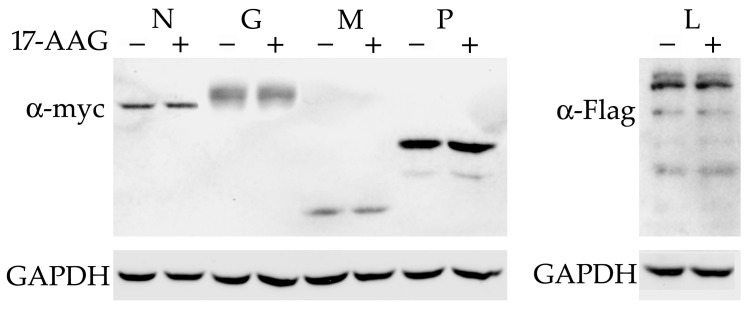
Hsp90 inhibition does not influence the expression of RABV proteins. HEK293 cells were transfected with plasmids expressing myc-tagged N, G, M, and P (**left** panel) and Flag-L (**right** panel). We added 500 nM 17-AAG 2 h after transfection. Expression was tested using anti-myc and anti-Flag antibodies 24 h later. The experiment was repeated twice, with similar results.

All the RABV proteins were cloned with myc or Flag-tags in the expression vectors. The experiments with plasmid-transfected N2a and NAC1300 cells showed that the expression of RABV genes was low. Therefore, the experiments that required the use of plasmids for the expression of RABV proteins were carried out using HEK293 cells. These cells are as effective as N2a cells in supporting RABV replication and effectively express proteins cloned under the control of CMV promoter [17]. The plasmids were transfected into HEK293 cells and 17-AAG was added 2 h later. The cells were lysed 24 h after transfection. The Western blot did not detect a decreased level of any RABV protein after 17-AAG treatment (Figure 2).

Previously, it was shown that the Hsp90 inhibitor decreased the expression of the P protein of the rabies virus strain HEP-Flury [16]. Here, we demonstrate that CVS-11 P protein expression is not affected by 17-AAG. To test whether the differences in the amino acid composition of these proteins may explain the difference in their dependence on Hsp90, we compared known sequences of RABV P proteins (Figure S2). CVS-11 and HEP-Flury P proteins are 95% identical. There are 15 different residues in these 297-amino-acid-long proteins. Many of the different residues substantially modify the properties of the proteins, changing folding (S_179_P), charge (Q_55_R, G_138_R, G_172_R, E_231_K, and K_295_T), or polarity (S_63_L and A_183_S). Alignment to other known P proteins did not reveal mutations unique to HEP-Flury or CVS-11, except for K_295_ in one of two existing alternative HEP-Flury sequences, which is replaced by A or T in other sequences. We did not find a sequence from a field virus identical to HEP-Flury, but two sequences differ from it by only one residue (ADK90892 and ACI01063) (Figure S3) [18].

### 2.3. RABV L Protein Is Expressed and Remains Soluble without the Presence of P Protein

**Figure 3:**
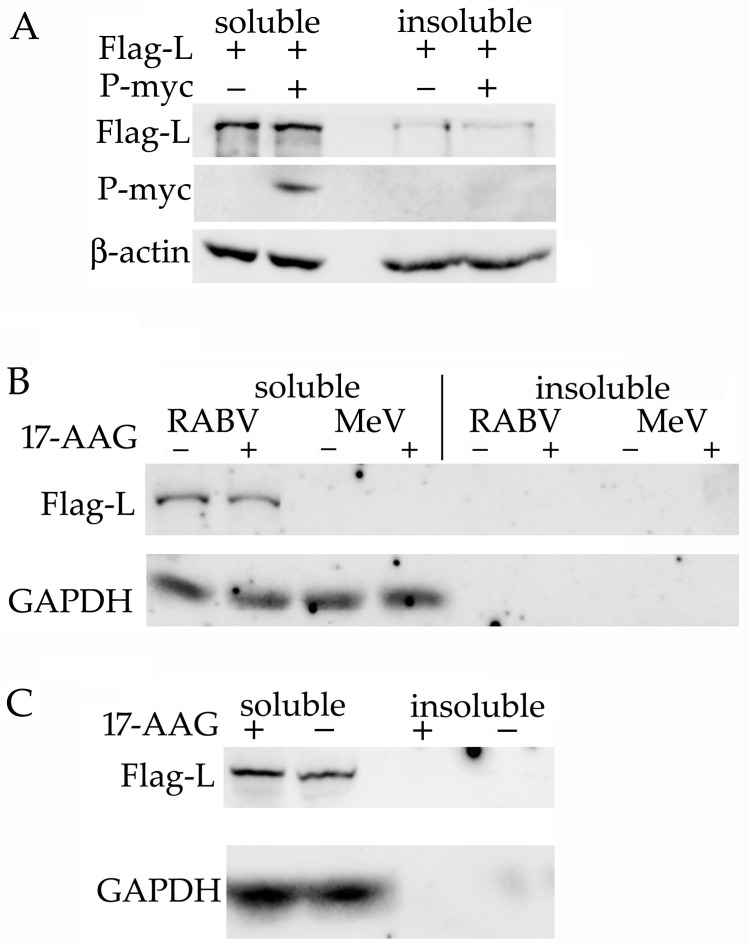
Soluble RABV L protein is expressed without P protein present. (**A**) HEK293 cells were transfected with plasmids that expressed the RABV Flag-L and P-myc proteins, as indicated. The cells were lysed with IP buffer 48 h after transfection. The insoluble fraction was separated by centrifugation and solubilized with 8 M of urea. Proteins were detected with antibodies specific for Flag and myc. HEK293 cells were transfected with plasmids that expressed the Flag-L proteins of the RABV and MeV virus (**B**) or with the plasmid that expressed VSV Flag-L (**C**). We added 0.5 μM 17-AAG 8 h after transfection. Cells were lysed 48 h after transfection. The results of one of two experiments are presented.

MuV P protein is necessary for L protein expression [14], and MeV L protein remains soluble only when co-expressed with P protein [13]. Therefore, we tested whether RABV L protein can be expressed in a soluble form without the presence of P protein. The lysates made of HEK293 cells that expressed L protein alone or co-expressed L and P proteins were separated into soluble and insoluble fractions by centrifugation. L protein alone was expressed at a similar level to L protein co-expressed with P protein, and most of the L protein remained soluble even in the absence of P protein. P protein was detected only in the soluble fraction (Figure 3A). To test the possibility that RABV L protein expression is a plasmid-specific artifact, we made plasmids with the RABV sequence replaced with the L genes of MeV and VSV. We did not detect the expression of the soluble or the insoluble MeV Flag-L protein (Figure 3B), but the VSV Flag-L protein was expressed and was detected only in the soluble fraction (Figure 3C).

### 2.4. Hsp90 Is Required for the L and P Proteins Association

**Figure 4:**
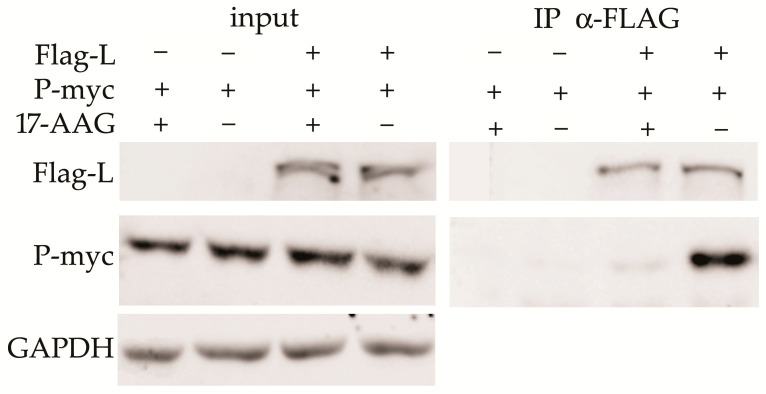
Hsp90 facilitates L–P complex formation. HEK293 cells were transfected with the P-myc and Flag-L plasmids as indicated. 17-AAG was added 2 h after transfection and the cells were lysed in IP buffer 24 h later. Agarose-bound α-Flag antibody was used for immunoprecipitation, and anti-Flag and anti-myc antibodies were used to detect precipitated proteins. The results of one of two experiments are presented.

HEK293 cells were transfected with the plasmids that express Flag-L and P-myc proteins. 17-AAG was added 2 h after transfection and cells were lysed 24 h later. Co-immunoprecipitation (Co-IP) was performed with anti-Flag agarose-bound antibody. The L-P protein complex was detected in control cells, but not in the cells exposed to 17-AAG (Figure 4).

### 2.5. 17-AAG Does Not Inhibit RABV RNA Synthesis Early after Infection

**Figure 5:**
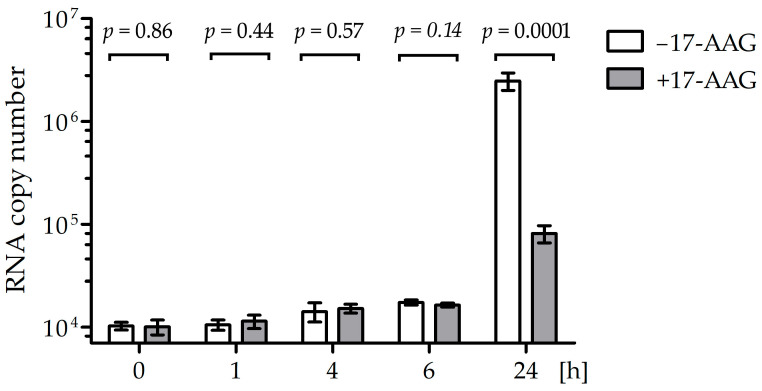
Right after infection, CVS-11 RNA synthesis is not affected by Hsp90 inhibition. NAC1300 cells pre-incubated for 2 h with 500 nM 17-AAG and control cells not treated with the inhibitor were infected on ice with CVS-11 at MOI = 100. After 1 h, unbound virus was washed off with cold PBS. The washed cells were incubated in a medium with or without 17-AAG. RNA was isolated at the indicated time and RABV RNA copies for the N gene were measured using RT-qPCR. The means and SD values of the four repeats of the experiment are plotted. The Student’s *t*-test was used to calculate the *p* values.

**Figure 6:**
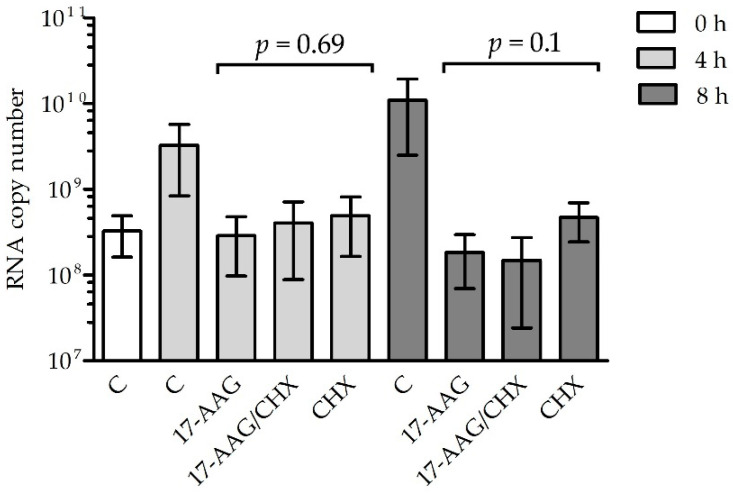
N2a cells were infected with CVS-11 at MOI = 10 for 1 h. The virus was washed off and the cells were incubated for 23 h. At this point, the medium was replaced with fresh medium supplemented with 50 μg/mL CHX and 1 μM 17-AAG (0 h), as indicated, and the incubation was continued for 4 or 8 h. The cells cultured in drug-free medium served as the control (C). Viral RNA was measured at the time of the addition of 17-AAG and CHX and after incubation with drugs in total cellular RNA by RT-qPCR and expressed as the number of N gene copies/μg of total RNA. The experiment was performed in triplicate. Statistical analysis was performed using one-way ANOVA.

The RNA polymerase contained in RABV particles should be active in the presence of 17-AAG if Hsp90 is necessary for the formation of the L–P complex, but not for the stability of the matured polymerase. To test this hypothesis, we measured the RABV RNA synthesis rate in cells early after infection (Figure 5). There was no significant difference in the mRNA quantity in cells treated with 17-AAG and the controls up to 6 h after infection, but 24 h after infection, the inhibition of viral RNA synthesis was clearly visible. To further prove that Hsp90 activity is necessary for the assembly of the RABV polymerase complex but not for the activity of the mature enzyme, we tested whether the 17-AAG treatment decreased viral RNA synthesis when de novo protein synthesis was inhibited by cycloheximide (CHX). Both 17-AAG and CHX decreased RABV replication, but we did not see a statistically significant difference in the RABV polymerase activity of cells treated with 17-AAG, CHX, and both drugs (Figure 6). This indicated that 17-AAG did not inhibit the mature polymerase that was present in the cells before new polymerase synthesis was inhibited by CHX.

## 3. Discussion

Xu et al. reported an unusually high expression of Hsp90 and its co-chaperone Cdc37 in N2a cells infected with the RABV HEP-Flury strain [16]. The elevated expression of these proteins was observed in proliferating cells, in cancers, or during tissue regeneration [19,20,21,22]. However, despite the universal dependence of virus replication on Hsp90 activity, we did not find other examples of the induced expression of Hsp90 or its co-chaperones in virus-infected cells. Therefore, we decided to test the effect of RABV CVS-11 infection on Hsp90 and Cdc37 expression in N2a and NAC1300 cells.

Xu et al. used a high virus concentration for infection (MOI = 1). We tested the results of infection at a high and low multiplicity of infection (MOI = 1 and 0.1) because the negative effect a high infection load on the RABV replication was noticed before, and the best viral yield was obtained with the infection at MOI = 0.1–0.3 [23,24]. We did not detect any Hsp90 or Cdc37 induction, regardless of the RABV concentration. The discrepancy between our results and the results reported earlier might be attributed to the RABV strain-specific differences. Xu et al. used the attenuated RABV strain HEP-Flury, which was selected during the vaccine development, whereas in our experiment, the pathogenic strain CVS-11 was used. CVS-11 is a highly virulent RABV strain, able to kill mice after intracerebral or periferal infection, whereas the less pathogenic HEP-Flury RABV strain does not cause fatal infection by either intracerebral or peripheral inoculations, suggesting that these viruses exhibit less in vivo neuroinvasiveness and lower in vitro neurotropism [25,26]. There are several potential mechanisms of Hsp90 influence on the replication of different strains of RABV. RABV infection induces type I interferon (IFN) production within the infected host cells [27]. In the neuronal cytoplasm, Hsp90 activity is necessary for phosphorylation and the nuclear transport of interferon regulatory factor 3 triggered by a viral RNA, a process that leads to the increased transcription of interferon α/β [28]. The wild-type RABV, but not HEP-Flury, infection leads to increased autophagy, which is also Hsp90-dependent [29,30]. The possibility of a RABV strain-specific regulation of Hsp90 and Cdc37 expression deserves further study, especially to correlate the results obtained for the laboratory RABV strains, such as HEP-Flury and CVS-11, and the field RABV isolates.

A previously published study of Hsp90 influence on RABV replication pointed at P protein as the most likely subject of Hsp90 chaperone [16]. This study reported that Hsp90 inhibition resulted in the degradation of HEP-Flury P, but not the N protein expressed from a plasmid in N2a cells. Here, we demonstrated that none of the RABV proteins expressed alone in HEK293 cells were affected by Hsp90 inhibition. In particular, the expression level of P protein did not decrease as a result of Hsp90 inactivation. The known examples of single-amino-acid-substitutions that change a mutant protein interaction with Hsp90 suggest that mutations in HEP-Flury P protein may decrease its stability and make it more dependent on Hsp90 chaperone activity [31,32,33]. An analysis of known P protein sequences demonstrated that amino acid differences in RABV HEP-Flury compared to CVS-11 are common in the field isolates of RABV and that the P proteins of some field and HEP-Flury viruses are nearly identical. Therefore, it is possible that both Hsp90-dependent P protein stability and/or Hsp90-dependent P–L complex formation may be responsible in different RABVs for the sensitivity to Hsp90 inhibition, depending on the P protein sequence.

The specific role of Hsp90 activity in the process of mononegaviruses replication was a subject of several other studies. These studies demonstrated that Hsp90 is involved in maintaining the stability of L protein and the L–P protein complex, but the exact function of Hsp90 seemed to be virus-specific. Hsp90 inhibition resulted in the degradation of the L protein of the respiratory syncytial viruses, Nipah (NiV), VSV, and MuV [13,14,15,34]. The dependence of L protein expression and stability on P protein co-expression was also reported [13,14,35]. P protein is necessary to prevent the L protein aggregation of MeV, VSV, NiV, and human parainfluenza virus 3 [13,36]. However, Hsp90 inhibition caused MeV L protein degradation only in the presence of P protein, whereas the degradation of NiV and VSV L protein occurred even in the absence of P protein. Here, we demonstrated that RABV L protein is expressed in its soluble form without the presence of P protein and that Hsp90 inhibition does not result in the decreased expression of RABV L protein. We did not detect MeV L protein, which may indicate that this protein is either not expressed or is cleared from the cytoplasm by degradation when its expression is low. The insoluble aggregates of MeV L may form only when the very efficient expression system is used [13]. The same may be true for VSV L protein, which was expressed from our plasmid in a soluble form independent of Hsp90 activity but was reported earlier to aggregate in an Hsp90-dependent manner when expressed at a high level [13]. The successful reconstitution of the active VSV polymerase in the mixed lysates of cells that separately expressed L and P proteins confirmed that at least some of the L protein of this virus expressed alone acquired native conformation and was ready to form an active complex with the P protein [37]. In agreement with these observations, the results of our study indicate that the proper folding and stability of RABV L protein does not depend on the activity of Hsp90 or on the complex formation of L and P proteins. However, the Co-IP results indicate that Hsp90 chaperone activity is necessary for the formation of the L–P proteins complex. This indicates that mature RNA polymerase L–P complex activity may be Hsp90-independent. Our finding that Hsp90 inhibition does not affect RABV RNA synthesis rate in the first 6 h after infection supports this model. Early after-infection RNA synthesis of the virus is catalyzed exclusively by the polymerase that was contained in the virus. This enzyme does not require Hsp90 chaperoning activity and is not sensitive to the presence of Hsp90 inhibitor. The difference in the virus RNA synthesis in cells treated and not treated with 17-AAG becomes apparent later, when newly translated P and L proteins cannot form active enzymes in cells treated with Hsp90 inhibitor. This model is also supported by the results of the experiment in which synthesis of the new P and L proteins was inhibited with CHX 24 h after infection. The polymerase present in the cells before CHX was added remained active when the cells were exposed to 17-AAG. This clearly demonstrates that mature RABV polymerase activity is Hsp90-independent.

P protein binding elicits a large structural rearrangement in L protein, a process that may require assistance from the Hsp90 chaperone [38]. The studies of the polymerases of other mononegaviruses and our results point at L protein as an Hsp90 subject, with the possible involvement of P protein in L protein maturation and stabilization. The properties of RABV L protein and its interaction with P protein and Hsp90 seem to be distinct from the L proteins of other mononegaviruses. The virus-specific differences in the structure of L protein and differences in the composition of L and P protein complexes may account for the differences in Hsp90’s role in mononegaviruses L protein maturation and L–P protein complex formation [12].

## 4. Materials and Methods

### 4.1. Cell Lines and Virus

The mouse neuroblastomas NAC1300 and NACCL-131 (N2a) and human embryonic kidney cell line HEK293 were purchased at American Type Culture Collection (ATCC). The NAC1300 and N2a cells were cultured in Eagle’s Minimum Essential Medium (ATCC) supplemented with 10% fetal bovine serum (FBS) (Gibco) and antibiotic-antimicotic solution (Sigma). The HEK293 cells were cultured in RPMI medium supplemented with 10% FBS and a penicillin-streptomycin mix (Gibco).

The rabies virus strain CVS-11 (laboratory strain of Challenge Virus Standard) was obtained from the European Reference Laboratory for Rabies (ANSES, Malzeville, Nancy, France) and was propagated in NAC1300 and N2a cell lines. The virus stocks were established at titres of 10^8.17^ TCID_50_/mL and 10^7.83^ TCID_50_/mL, respectively.

### 4.2. In Vitro Assay

The HEK293, NAC1300, and N2a cells were seeded in 24-well plates at 160,000 cells/well. After 24 h, cells were infected for 1 h with 100 or 1000 TCID_50_ of CVS-11 in 50 μL medium (MOI = 0.1 and 1, respectively). After that, the inoculum was replaced with medium supplemented with different concentrations of 17-AAG suspended in Eagle’s Minimum Essential Medium with 5% of FBS, which were added to the appropriate wells. The cells were incubated for 48 h and then the supernatants from each of the wells were collected and stored at −80 °C until the virus titration and RT-qPCR assay, whereas the cell monolayer was subjected to lysis using an RIPA buffer.

### 4.3. Virus Titration

Using a fluorescent focus assay, 10-fold dilutions of the supernatants were titrated on the NAC1300 and N2a cells in quadruplicates [39]. The titration plates were incubated for 48 h, fixed with 80% acetone for 20 min, and stained with anti-rabies FITC-labeled monoclonal antibody (Fujirebio Diagnostics, Malvern, PA, USA). Fluorescence was evaluated using an inverted microscope under UV light. The RABV titres were calculated using the Spearmann–Kärber method and expressed in a 50% tissue culture infectious dose (TCID_50_/mL) [39].

### 4.4. Cell Viability Assay

Approximately 80% of the confluent cells were incubated with different concentrations of 17-AAG for 48 h, and viability was measured using the CellTiter-Glo assay (Promega).

### 4.5. Reverse Transcription Quantitative PCR (RT-qPCR)

The total RNA was extracted from the supernatants using a QIAamp Viral RNA Mini Kit (Qiagen, Hilden, Germany) and from the cell monolayers using Tri Reagent (Merck, MA, USA). *RT-qPCR* was performed using a QuantiTect Probe RT-PCR Kit (Qiagen, Hilden, Germany), with the primers listed in Table 1, based on 10-fold dilutions of the RABV standard [40].

### 4.6. Plasmids Construction

The RNA isolated from the NAC1300 cells infected with the CVS-11 virus was subjected to reverse transcription using an oligo-dT primer. The coding sequences of the genes were amplified by PCR with the primers listed in Table 1 and were cloned into the plasmid pcDNA-myc to create C-terminal fusions with the myc-tag sequence using the sequences for restriction enzymes incorporated in the sequence of primers [41]. Plasmid pCMV-10 was used to clone the L CVS-11 gene as an N-terminal fusion with the 3xFlag sequence, and the resulting plasmid was named pWSL5. The plasmids with cloned MeV and VSV L proteins were used as templates to amplify the DNA fragments that were used to replace the CVS-11 L gene in the pWSL5 [13,42]. The PCR product of the pWSL5 amplification with the primers pWSL5-F and pWSL5-R was used to make the plasmids containing the 3xFLAG fusions of the MeV and VSV L proteins. These plasmids were constructed using PCR products amplified with MeV L- and VSV L-specific primers using the cohesive ends generated with T4 polymerase [43].

### 4.7. Western Blot Analysis

Transfection with plasmids that expressed RABV proteins was performed using Metafectane reagent, following the manufacturers’ instructions (Biontex). We added 500 nM 17-AAG to the transfected cells 6 h after infection, and the incubation was terminated 48 h after transfection. The protein extracts prepared with the RIPA buffer were resolved on 10% PAGE-SDS gels to detect myc-tagged G, M, N, and P proteins. 3xFLAG-L protein was resolved on 8% PAGE-SDS gel. The insoluble protein was separated by centrifugation for 20 min at 14,000× *g* and the pellet was solubilized in 8M urea before electrophoresis. The proteins were detected using monoclonal antibodies specific for Flag (Sigma, F3165) and myc (Merck, MABE282). A goat anti-mouse IgG-HRP antibody was obtained from Bio-Rad (cat. no. 170-6516). The original pictures were taken with a CCD camera with the exposure time adjusted to avoid pixel saturation. The registered signals were within the linear response range of the camera.

### 4.8. Co-Immunoprecipitation (Co-IP)

For Co-IP, HEK293 cells were transfected with P-myc and 3xFlag-L or P-myc alone plasmids. We added 500nM 17-AAG 2 h after transfection. After 24 h, the cells were harvested and lysed with IP buffer (0.25% Triton X-100, 10 mM Tris-HCl pH 7.5, 20 mM NaF, 100 mM ATP, 10 mM β-glycerol phosphate, 2 mM sodium orthovanadate, and protease inhibitors cocktail (Roche)). The lysates were cleared by centrifugation at 12,000× *g* for 15 min at 4 °C. The protein concentration was measured using the BCA assay (Sigma) and adjusted with IP buffer to 1 mg/mL. We added 10 μL anti-Flag agarose beads (Sigma) to the supernatant (700 μL) and then incubated for 2 h at 4 °C with mixing. The immunoprecipitates were washed with ice-cold PBS four times and eluted with 40 μL 1× SDS PAGE Loading buffer. The samples were boiled for 10 min and analyzed by Western blot.

### 4.9. Sequence and Phylogenetic Analysis

The GeneBank database was searched using the amino acid sequence of the RABV HEP-Flury P protein. The one-hundred most similar sequences were aligned and analyzed with BioEdit v.7 software [44]. Phylogenetic analysis was performed using MEGA11 software [45].

## Figures and Tables

**Table 1:** Primers used for cloning. The KpnI and NotI sites used for plasmid construction are underlined.

N-forward	GCTCTGGTACCATGGATGCCGACAAGATTG
N-reverse	CGCGAGCGGCCGCAATGAGTCATTCGAATACGTCTTG
P-forward	GCTCTGGTACCATGAGCAAGATCTTTGTTAATCC
P-reverse	CGCGAGCGGCCGCAAGCAGGATGTATAGCGATTC
G-forward	GCTCTGGTACCATGGTTCCTCAGGTTCTTTTG
G-reverse	CGCGAGCGGCCGCCACAGTCTGATCTCACCTCC
M-forward	GCTCTGGTACCATGAACGTTCTACGCAAGATAG
M-reverse	CGCGAGCGGCCGCAATTCTAGAAGCAGAGAAGAGTC
RABV L-forward	CGACTGGTACCATGCTAGATCCGGGAGAGGT
RABV L-reverse	CGCGAGCGGCCGCAACAAACAACTGTAATCTAGTAGG
MeV L-F	ATAGATCTGGATATCGGTACCATGGACTCGCTATCTGTCAAC
MeV L-R	CTAGAAGGCACAGTCGAGGCTTAGTCCTTAATCAGGGCACTG
VSV L-F	ATAGATCTGGATATCGGTACCATGGAAGTCCACGATTTTGAGAC
VSV L-R	CTAGAAGGCACAGTCGAGGCTTAATCTCTCCAAGAGTTTTCCTCG
pWSL5-F	GCCTCGACTGTGCCTTCTAG
pWSL5-R	GGTACCGATATCCAGATCTATCGA
qPCR primer F	ATGTAACACCTCTACAATG
qPCR primer R	GCAGGGTATTTRTACTCATA
qPCR probe	ACAAGATTGTATTCAAAGTCAATAATCAG

## Data Availability

The data that support the findings of this study are available from the corresponding author upon reasonable request.

## Supplementary Materials

The following are available online at https://www.mdpi.com/article/10.3390/ijms23136946/s1.
