# Supplementary material for: Hsp90 Activity Is Necessary for the Maturation of Rabies Virus Polymerase
Source: Int J Mol Sci. 2022 Jun 22;23(13):6946. doi: 10.3390/ijms23136946 (PMC9266396; doi:10.3390/ijms23136946)
Supplement: Supplementary file 1 [file ijms-23-06946-s001.zip › ijms-1726119-supplementary.pdf]

Supplementary Figure S1.

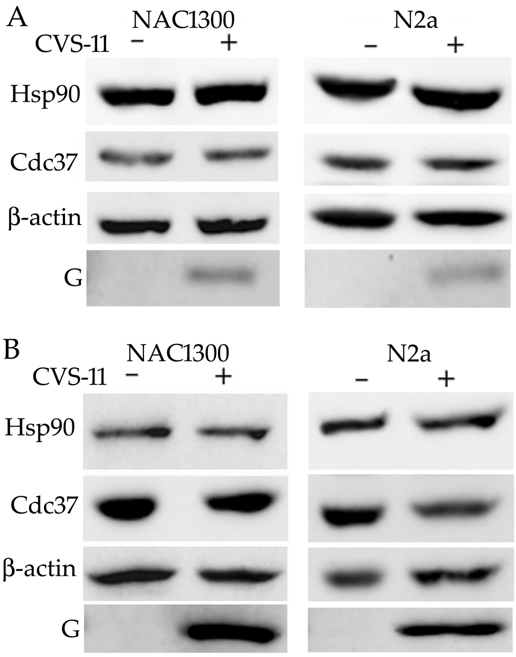

Hsp90 and Cdc37 expression is not elevated in RABV infected cells. NAC1300 and N2a cells were infected with CVS-11 at MOI=0.1 (A) and MOI=1 (B) titre. Hsp90 and Cdc37 expression in the infected and not infected cells was compared 48 h after infection. GAPDH and RABV Glycoprotein (G) were used as control for protein load and virus infection. The experiment was repeated twice with the similar results.

Supplementary Figure S2

```
                   10        20        30        40        50        60        70        80        90
           ....|....|....|....|....|....|....|....|....|....|....|....|....|....|....|....|....|....|
P22363     MSKIFVNPSAIRAGLADLEMAEETVDLINRNIEDNQAHLQGEPIEVDNLPEDMKRLHLDDEKSSNLGEMVRVGEGKYREDFQMDEGEDPN
Q8B6J8     MSKIFVNPSAIRAGLADLEMAEETVDLINRNIEDNQAHLQGEPIEVDNLPEDMRQFHLDDEKLSNLGEMVRVGEGKYREDFQMNEGEDPN
ADD84788   MSKIFVNPSAIRAGLADLEMAEETVDLINRNIEDNQAHLQGEPIEVDNLPEDMRQFHLDDEKLSNLGEMVRVGEGKYREDFQMNEGEDPN
AAK54990   MSKIFVNPSAIRAGLADLEMAEETVDLINRNIEDNQAHLQGEPIEVDNLPEDMRQFHLDDEKLSNLGEMVRVGEGKYREDFQMNEGEDPN
AAK55055   MSKIFVNPSAIRAGLADLEMAEETVDLINRNIEDNQAHLQGEPIEVDNLPEDMRQFHLDDEKLSNLGEMVRVGEGKYREDFQMNEGEDPN
ADK90925   MSKIFVNPSAIRAGLADLEMAEETVDLINRNIEDNQAHLQGEPIEVDNLPEDMRQFHLDDEKLSNLGEMVRVGEGKYREDFQMNEGEDPD
ACI01063   MSKIFVNPSGIRAGLADLEMAEETVDLINRNIEDNQAHLQGEPIEVDNLPEDMRQFHLDDEKLSNLGEMVRVGEGKYREDFQMNEGEDPN
ADK90892   MSKIFVNPSAIRAGLADLEMAEETVDLINRNIEDNQAHLQGEPIEVDNLPEDMRQFHLDDEKLSNLGEMVRVGEGKYREDFQMNEGEDPN
ACL98055   MSKIFVNPSAIRAGLADLEMAEETVDLINRNIEDNQAHLQGEPIEVDNLPEDMRQFHLGDEKLSNLGEMVRVGEGKYREDFQMDEGEDPN
ABV64427   MSKIFVNPSAIRAGLADLEMAEETVDLINRNIEDNQAHLQGEPIEVDNLPEDMRQFHLDDEKLSNLGEMVRVGEGKYREDFQMNEGEDPN
AAZ07887   MSKIFVNPSAIRAGLADLEMAEETVDLINRNIEDNQAHLQGEPIEVDNLPEDMRQFHLDDEKLSNLGEMVRVGEGKYREDFQMDEGEDPN
CAA40929   MSKIFVNPSAIRAGLADLEMAEETVDLINRNIEDNQAHLQGEPIEVDNLPEDMKRLHLDDEKSSNLGEMVRVGEGKYREDFQMDEGEDPN
AAK54997   MSKIFVNPSAIRAGLADLEMAEETVDLINRNIEDNQAHLQGEPIEVDNLPEDMRRLHLDDERSPNLGEMARVGEGKYREDFQMDEGEDPN
AEK98547   MSKIFVNPSAIRAGLADLEMAEETVDLINRNIEDNQAHLQGEPIEVDNLPEDMKRLHLDDEKSSNLGEMVRVGEGKYREDFQMDEGEDPN
P15198     MSKIFVNPSAIRAGLADLEMAEETVDLINRNIEDNDAHLQGEPIEVDNLPEDMKRLHLDDEKSSNLGEMVRVGEGKYREDFQMDEGEDPN
AFN27424   MSKIFVNPRAIRAGLADLEMAEETVDLINRNIEDNQAHLQGEPIEVDNLPEDMKRLHLDDEKSSNLGEMVRVGEGKYREDFQMDEGEDPN
BBB16467   MSKIFVNPSAIRAGLADLEMAEETVDLINRNIEDNQAHLQGEPIEVDNLPEDMKRLHLDDEKSSNLGEMVRVGEGKYREDFQMDEGEDPN
AAZ07892   MSKIFVNPSAIRAGLADLEMAEETVDLINQNIEDNQAHLQGEPIEVDNLPEDMKRLHLDDEKSSNLGEMVRVGEGKYREDFQMGEGEDPN
AAK55053   MSKIFVNPSAIRAGLADLEMAEETVDLINRNIEDNQAHLQGEPIEVDNLLEDMKRLHLDDEKSSNLGEMVRVGEGKYREDFQMDEGEDPN
APD76552   MSKIFVNPSAIRAGLADLEMAEETVDLINRNIEDNQAHLQGEPIEVDNLPEDMRRLHLDDEKSSNLGEMANVGEGRYREDFQMDEGEDPN
Q0GBY3     MSKIFVNPSAIRAGLADLEMAEETVDLINRNIEDNQAHLQGEPIEVDDLPEDMKRLHLDDEKSSNLGEMVRVGEGKYREDFQMDEGEDPN
SOF05866   MSKIFVNPSAIRAGLADLEMAEETVDLINRNIEDNQAHLQGEPIEVDNLPEDMRRLHLDDEKSSNLGEMANVGEGRYREDFQMDEGEDPN
AFN24401   MSKIFVNPSAIRAGLADLEMAEETVDLINRNIEDNQAHLQGEPIEVDNLPEDMRRLHLDDEKSSNLGEVVRAGEGKYREDFQMDEGEDPN
APD76597   MSKIFVNPSAIRAGLADLEMAEETVDLINRNMEDNQAHLQGEPIEVDNLPEDMRRLHLDDEKSSNLGEMANVGEGRYREDFQMDEGEDPN
ABF82951   MSKIFVNPSAIRAGLADLEMAEETVDLINRNIEDNQAHLQGEPIEVDNLPEDMKRLHLDDEKSSNLGEMVRVGEGKYREDFQMDEGEDPN
ASU55987   MSKIFVNPSAIRAGLADLEMAEETVDLINRNIEDNQAHLQGEPIEVDNLPEDMRRLHLDDEKSSNLGEMANVGEGKYREDFQMDEGEDPN
AAY32477   MSKIFVNPSAIRAGLADLEMAEETVDLINRNIEDNQAHLRGEPIEVNNLPEDMKRLHLDDEKSSNLGEMVRVGEGKYREDFQMDEGEDPN
APD76602   MSKIFVNPSAIRAGLADLEMAEETVDLINRNIEDNQAHLQGEPIEVDNLPEDMRRLHLDDEKSSNLGEMANVGEGKYREDFQMDEGEDPN
ADU55579   MSKIFVNPSAIRAGLADLEMAEETVDLINRNIEDNQAHLQGEPIEVDNLPEDMRRLHLDDEKPSSLGEMANVGEGKYREDFQMDEGEDPN
ALC74030   MSKIFVNPSAIRAGLADLEMAEETVDLINRNIEDNQAHLQGEPIEVDNLPEDMRRLHLDDEKSSNLGEMANVGEGKYREDFQMDEGEDPN
AAK55039   MSKIFVNPSAIRAGLADLEMAEETVDLINRNIEDNQAHLQGEPIEVDNLPEDMRRLHLDDEKSSNLGEMANVGEGRYREDFQMDEGEDPN
AAK55015   MSKIFVNPSAIRAGLADLEMAEETVDLINRNIEDNQAHLQGEPIEVDNLPEDMRRLHLDDEKSSNLGEMANVGEGKYREDFQMDEGEDPN
ASU55977   MSKIFVNPSAIRAGLADLEMAEETVDLINRNIEDNQAHLQGEPIEVDNLPEDMRRLHLDDEKSSNLGEMANVGEGKYREDFQMDEGEDPN
AAK55034   MSKIFVNPSAIRAGLADLEMAEETVDLINRNIEDNQAHLQGEPIEVDNLPEDMRRLHLDDEKSSNLGEMDNVGEGKYREDFQMDEGEDPN
AFN24256   MSKIFVNPSAIRAGLADLEMAEETVDLINRNIEDNQAHLQGEPIEVDSLPEDMRRLHLGDEKSSNLGEMAKVGEGKYREDFQMDEGEDPN
AUT19348   MSKIFVNPSAIRAGLADLEMAEETVDLINRNIEDNQAHLQGEPIEVDNLPEDMRRLHLDDGKSSNLGEMAKAGEGKYREDFQMDEGEDPS
AAK55048   MSKIFVNPSAIRAGLADLEMAEETVDLINKNIEDNQAHLQGEPIEVDNLPEDMRQLHLDDGKSSNLGEMAKVGEGKYREDFQMDEGEDPS
AUT19448   MSKIFVNPSAIRAGLADLEMAEETVDLINRNIEDNQAHLQGEPIEVDNLPEDMRRLHLDDGKSSNLGEMAKAGEGKYREDFQMDEGEDPS
APD76997   MSKIFVNPSAIRAGLADLEMAEETVDLINRNIEDNQAHLQGEPIEVDNLPEDMRRLHLDDEKSSNLNEMAKAGEGKYREDFQMDEGEDPS
AUT19373   MSKIFVNPSAIRAGLADLEMAEETVDLINRNIEDNQAHLQGEPIEVDNLPEDMRRLHLDDGKSSNLGEMAKAGEGKYREDFQMDEGEDPS
AAK55042   MSKIFVNPSAIRAGLADLEMAEETVDLINRNIEDNQAHLQGDPIEVDNLPEDMKRLHLDDEKSSNLGVMVRVEKSKYREDFQMDEGEDPN
QEU57682   MSKIFVNPSAIRAGLADLEMAEETVDLINRNIEDNQAHLQGEPIEVDNLPEDMRRLHLEDGKSSNLGEMAKAGEGKYREDFQMDEGEDPS
QDZ26173   MSKIFVNPSAIRAGLADLEMAEETVDLINKNIEDNQAHLQGEPIEVDNLPEDMRRLHLDDGKSSNLGEMAKAGEGKYREDFQMDEGEDPS
AUT19463   MSKIFVNPSAIRAGLADLEMAEETVDLINRNIEDNQAHLQGEPIEVDNLPEDMRRLHLDDGKSSNLSEMTRAGEGKYREDFQMDEGEDPS
QDF46328   MSKIFVNPSAIRAGLADLEMAEETVDLINKNIEDNQAHLQGEPIEVDNLPEDMRQLHLDDGKSSNLGEMAKVGEGKYREDFQMDEGEDPS
APD76752   MSKIFVNPSAIRAGLADLEMAEETVDLINRNIEDNQAHLQGEPIEVDNLPEDMRRLHLDEGKSSNLGEMAKAGEGKYREDFQMDEGEDPS
AAK55049   MSKIFVNPSAIRAGLADLEMAEETVDLINRNIEDNQAHLQGEPIEVDNLPEDMRRLHLDDEKSSNLGEMAKVGEDKYREDFQMDEGEDPS
AUT19488   MSKIFVNPSAIRAGLADLEMAEETVDLINRNIEDNQAHLQGEPIEVDNLPEDMRRLHLDDGKSSNLSEMTRAGEGKYREDFQMDEGEDPS
AAK55004   MSKIFVNPSAIRAGLADLEMAEETVDLINRNIEDNQAHLQGEPIEVDNLPEDMRRLHLDDGKSSNLGEMAKAGEGKYREDFQMDEGEDPS
ARM53424   MSKIFVNPSAIRAGLADLEMAEETVDLINKNIEDNQAHLQGEPIEVDNLPEDMRRLHLDDGKSSNLCEMVNAGEGKYREDFQMNEGEDPN
APD76857   MSKIFVNPSAIRAGLADLEMAEETVDLINRNIEDNQAHLQGEPIEVDNLPEDMRRLHLDDGKSSSLGEMAKSGEGKYREDFQMDEGEDPS
CUI02212   MSKIFVNPSAIRAGLADLEMAEETVDLINKNIEDNQAHLQGEPIEVDNLPEDMRRLHLDDGKSANLGEMAKAGEGKYREDFQMDEGEDPS
APD76852   MSKIFVNPSAIRAGLADLEMAEETVDLINRNIEDNQAHLQGEPIEVDNLPEDMRRLHLDDGKSSSLGEMAKSGEGKYREDFQMDEGEDPS
AUT19538   MSKIFVNPSAIRAGLADLEMAEETVDLINRNIEDNQAHLQGEPIEVDNLPEDMRRLHLDEGKSSNLGEMAKAGEGRYREDFQMDEGEDPS
AAK54999   MSKIFVNPSAIRAGLADLEMAEETVDLINRNIEDNQAHLQGEPIEVDNLPEDMRRLHLDDGKLSNLGEMAKAGEGKYREDFQMDEGEDPG
APD76757   MSKIFVNPSAIRAGLADLEMAEETVDLINRNIEDNQAHLQGEPIEVDNLPEDMRRLHLDEGKSSNLGEMAKAGEGKYREDFQMDEGEDPS
APD76927   MSKIFVNPSAIRAGLADLEMAEETVDLINRNIEDNQAHLQGEPIEVDNLPEDMRQLHLDDGKSSNLSEMVKAGEGKYREDFQMDEGEDPS
AUT19363   MSKIFVNPSAIRAGLADLEMAEETVDLINRNIEDNQAHLQGEPIEVDNLPEDMRRLHLDDGKSSNLGEMAKAGEGKYREDFQMDEGEDPS
ADR67361   MSKIFVNPSAIRAGLADLEMAEETVDLINKNIEDNQAHLRGEPIEVDNLPEDMRQLHLDDGKSSNLGEMAKVGEGKYREDFQMDEGEDPS
AUT19543   MSKIFVNPSAIRAGLADLEMAEETVDLVNRNIEDNQAHLQGEPIEVDSLPEDMRRLHLDDGKLSNLGEMAKAGEGKYREDFQMDEGEDPS
AUT19393   MSKIFVNPSAIRAGLADLEMAEETVDLINRNIEDNQAHLQGEPIEVDNLPEDMRRLHLDDGKSSNLGEMAKAGEGKYREDFQMDEGEDPS
APD77002   MSKIFVNPSAIRAGLADLEMAEETVDLINRNIEDNQAHLQGEPIEVDNLPEDMRRLHLDDEKSSNLNGMAKAGEGKYREDFQMDEGEDPS
AAK55038   MSKIFVNPSAIRAGLADLEMAEETVDLINRNIEDNQAHLQGEPIEVGNLPEDMRRLNLDDGKSSNLGEMVKAGEGKYREDFQMDEGEDPS
AFN24101   MSKIFVNPSAIRAGLADLEMAEETVDLINRNIEDNQAHLQGEPIEVDSLPEDMSRLHLGDEKPSNLGEMAKVGEGKYREDFQMDEGEDPN
APD76687   MSKIFVNPSAIRAGLADLEMAEETVDLVNRNIEDNQAHLQGEPIEVDNLPEDMRQLHLDDGKPSNLGEMAKAGEGKYREDFQMDEGEDPS
QEU57887   MSKIFVNPSAIRAGLADLEMAEETVDLVNRNIEDNQAHLQGEPIEVDNLPEDMRRLHLDDEKSSNLSEMAKTGEGKYREDFQMDEGEDPS
APD76932   MSKIFVNPSAIRAGLADLEMAEETVDLINRNIEDNQAHLQGEPIEVDNLPEDMRQLHLDDGKSSNLSEMVKAGEGKYREDFQMDEGEDPS
APD76872   MSKIFVNPSAIRAGLADLEMAEETVDLINRNIEDNQAHLQGEPIEVDNLPEDMRRLHLDDGKSSSLGEMAKSGEGKYREDFQMDEGEDPS
APD76847   MSKIFVNPSAIRAGLADLEMAEETVDLINRNIEDNQAHLQGEPIEVDNLPEDMRRLHLDDGKSSNLGEMAKAGEGKYREDFQMDEGEDPS
```

```
APD76867  MSKIFVNPSAIRAGLADLEMAEETVDLINRNIEDNQAHLQGEPIEVDNLPEDMRRLHLNDGKSSSLGEMAKSGEGKYREDFQMDEGEDPS
AAK55005  MSKIFVNPSAIRAGLADLEMAEETVDLINRNIEDNQAHLQGEPIEVDNLPEDMRRLHLDDGKSSNLGEMARAGEGKYREDFQMDEGEDPG
APD76702  MSKIFVNPSAIRAGLADLEMAEETVDLINRNIEDNQAHLQGEPIEVDNLPEDMRQLHLDDGKPSNLGEMAKAGEGKYREDFQMDEGEDPG
QEU57512  MSKIFVNPSAIRAGLADLEMAEETVDLVNRNIEDNQAHLQGEPIEVDNLPEDMRRLHLDDGKLSNLSEMAKTGEGKYREDFQMDEGEDPS
APD76742  MSKIFVNPSAIRAGLADLEMAEETVDLINRNIEDNQAHLQGEPIEVDNLPEDMRRLHLDEGKSSNLGEMAKAGEGRYREDFQMDEGEDPS
APD76767  MSKIFVNPSAIRAGLADLEMAEETVDLINRNIEDNQAHLQGEPIEVDNLPEDMRRLHLDEGKSSNLGGMAKAGEGKYREDFQMDEGEDPS
AUT19478  MSKIFVNPSAIRAGLADLEMAEETVDLINRNIEDNQAHLQGEPIEVDNLPEDMRRLHLDDGKSSSLGEMAKSGEGKYREDFQMDEGEDPS
AGQ16829  MSKIFVNPSAIRAGLADLEMAEETVDLINRNIEDNQAHLQGEPIEVDNLPEDMRRLNLDDGKSPNLGEIVRAGEGKYREDFQMDEGEDPS
APD76937  MSKIFVNPSAIRAGLADLEMAEETVDLINRNIEDNQAHLQGEPIEVDNLPEDMRQLHLDDGKSSNLSEMVKAGEGKYREDFQMDEGEDPS
AMX81156  MSKIFVNPSAIRAGLADLEMAEETVDLINRNIEDNQAHLQGEPIEVDNLPEDMRRLHLDDEKSSNLGEMSKTGEGKYREDFQMDEGEDPS
QEU57537  MSKIFVNPSAIRAGLADLEMAEETVDLINRNIEDNQAHLQGEPIEVDNLPEDMRRLNLDDGKSPNLGEIVKAGEGKYREDFQMDEGEDPS
AGQ16839  MSKIFVNPSAIRAGLADLEMAEETVDLINRNIEDNQAHLQGEPIEVDNLPEDMRRLHLDDGKSSNLGEMSKTGEGKYREDFQMDEGEDPS
AUT19388  MSKIFVNPSAIRAGLADLEMAEETVDLINRNIEDNQAHLQGEPIEVDNLPEDMRRLHLDDGKSSNLGEMAKAGESKYREDFQMDEGEDPS
CUI02217  MSKIFVNPSAIRAGLADLEMAEETVDLINRNIEDNQAHLQGEPIEVDNLPEDMRRLHLDDGRSSNLGEMAKAGEGKYREDFQMDEGEDPG
APD76697  MSKIFVNPSAIRAGLADLEMAEETVDLINRNIEDNQAHLQGEPIEVDNLPEDMRQLHLDDGKPSNLGEMAKAGEGKYREDFQMDEGEDPS
AMX81026  MSKIFVNPSAIRAGLADLEMAEETVDLINRNIEDNQAHLQGEPIEVDNLPEDMRRLHLDDGKSSNLGEMSKTGEGKYREDFQMDEGEDPS
AAK55001  MSKIFVNPSAIRAGLADLEMAEETIDLINRNIEDNQAHLQGEPIEVDNLPEDMRRLHLDDGKSSNLGEMSKTGEGKYREDFQMDEGEDPS
QEU57502  MSKIFVNPSAIRAGLADLEMAEETVDLINRNIEDNQAHLQGEPIEVDNLPEDMRRLNLDDGKSPNLGEIVKAGEGKYREDFQMDEGEDPS
APD76977  MSKIFVNPSAIRAGLADLEMAEETVDLINRNIEDNQAHLQGEPIEVDNLPEDMRRLHLDDGKSSNLSEMAKAGEGKYREDFQMDEGEDPS
SOF05896  MSKIFVNPSAIRAGLADLEMAEETVDLINRNIEDNQAHLQGEPIEVDNLPEDMRRLHLDDGKSSNLDEMVKAGEGKYREDFQMDEGEDPS
AUT19573  MSKIFVNPSAIRAGLADLEMAEETVDLINRNIEDNQAHLQGEPIEVDNLPEDMRRLHLDDGKSSNLGEMSNMGEGKYREDFQMDEGEDPS
AMX81051  MSKIFVNPSAIRAGLADLEMAEETVDLINRNIEDNQAHLQGEPIEVDNLPEDMRRLHLDDGKSSNLGEMSKTGEGKYREDFQMDEGEDPS
AMX80911  MSKIFVNPSAIRAGLADLEMAEETIDLINRNIEDNQAHLQGEPIEVDNLPEDMRQLHLDDGKSSNLGEMSKTGEGKYREDFQMDEGEDPS
APD76747  MSKIFVNPSAIRAGLADLEMAEETVDLINRNIEDNQAHLQGEPIEVDNLPEDMRRLHLDEGKSSNLGEMAKAGEGKYREDFQMDEGEDPS
AMA67989  MSKIFVNPSAIRAGLADLEMAEETVDLINRNIEDNQAHLQGEPIEVDNLPEDMRRLHLDDGKSSNLDEMVKAGEGKYREDFQMDEGEDPS
AMX81166  MSKIFVNPSAIRAGLADLEMAEETIDLINRNIEDNQAHLQGEPIEVDNLPEDMRRLHLDDGKSSNLGEMSKTGEGKYREDFQMDEGEDPS
APD76837  MSKIFVNPSAIRAGLADLEMAEETVDLINKNIEDNQAHLQGEPIEVDNLPEDMRRLHLDDGKSSNLGEMAKAGEGKYREDFQMDEGEDPS
AMA68025  MSKIFVNPSAIRAGLADLEMAEETVDLINRNIEDNQAHLQGEPIEVDNLPEDMRRLHLDDGKSSNLGEMSKTGEGKYREDFQMDEGEDPS
APD77102  MSKIFVNPSAIRAGLADLEMAEETVDLINRNIEDNQAHLQGEPIEVDNLPEDMRRLNLDDGKSPNLGELVKAGEGKYREDFQMDEGEDPT
AAK55060  MSKIFVNPSAIRAGLADLEMAEETVDLINRNIEDNQAHLQGEPIEVDNLPEDMRRLHLDDGKSSNLGEMAKVGEGKYREDFQMDEGEDPS
QKS69474  MSKIFVNPSAIRAGLADLEMAEETVDLINRNIEDNQAHLQGEPIEVDNLPEDMRRLHLDDGKSSNLGEMSETGEGKYREDFQMDEGEDPS
```

```
                    100       110       120       130       140       150       160       170       180
          ....|....|....|....|....|....|....|....|....|....|....|....|....|....|....|....|....|....|
P22363    LLFQSYLDNVGVQIVRQMRSGERFLKIWSQTVEEIVSYVTVNFPNPPRRSSEDKSTQTTGRELKKETTSAFSQRESQPSKARMVAQVAPG
Q8B6J8    LLFQSYLDNVGVQIVRQMRSGERFLKIWSQTVEEIISYVSVNFPNPPGRSSEDKSTQTTGRELKKETTSILSQRESQPSKAGMVAQVASG
ADD84788  LLFQSYLDNVGVQIVRQMRSGERFLKIWSQTVEEIISYVTVNFPNPPGRSSEDKSTQTTGRELKKETTSILSQRESQPSKAGMVAQVASG
AAK54990  LLFQSYLDNVGVQIVRQMRSGERFLKIWSQTVEEIISYVTVNFPNPPGRSSEDKSTQTTGRELKKETTSILSQRESQPSKAGMVAQVASG
AAK55055  LLFQSYLDNVGVQIVRQMRSGERFLKIWSQTVEEIISYVTVNFPNPPGRSSEDKSTQTTGRDLKKETTSILSQRESQPSKAGMVAQVASG
ADK90925  LLFQSYLDNVGVQIVRQMRSGERFLKIWSQTVEEIISYVSVNFPNPPGRSSEDKSTQTTGRELKKETTSILSQRESQPSKAGMVAQVASG
ACI01063  LLFQSYLDNVGVQIVRQMRSGERFLKIWSQTVEEIISYVSVNFPNPPGRSSEDKSTQTTGRELKKETTSILSQRESQPSKAGMVAQVASG
ADK90892  LLFQSYLDNVGVQIVRQMRSGERFLKIWSQTVEEIISYVSVNFPNPPGRSSEDKSTQTTGRELKKETTSILSQRESQPSKAGMVAQVASG
ACL98055  LLFQSYLDNVGVQIVRQMRSGERFLKIWSQTVEEIISYVTVNFPNPPGRSSEDKSTQTTGRELKKETTSTLSQRESQPSKAGMVAQVASG
ABV64427  LLFQSYLDNVGVQIVRQMRSGERFLKIWSQTVEEIISYVSVNFPNPPGRSSEDKSTQTTGRELKKETTSILSQRESQPSKAGMVAQVASG
AAZ07887  LLFQSYLDNVGVQIVRQMRSGERFFKIWSQTVEEIISYVTVNFPNPPGRSSEDKSTQTTGRELKKETTSTLSQRESQPSKAGMVAQVASG
CAA40929  LLFQSYLDNVGVQIVRQMRSGERFLKIWSQTIEEIVSYVTVNFPNPPRRSSEDKSTQTTGRELKKETTSAFSQRESQPSKARMVAQVAPG
AAK54997  LLFQSYLDNVGVQIVRQMRSGERFLKIWSQTVEEIISYVTVNFPNPSGRSSEDKSTQTTGRELKKETTSNLSQRESQPSKTRMVAQVASG
AEK98547  LLFQSYLDNVGVQIVRQMRSGERFLKIWSQTVEEIVSYVTVNFPNPPRRSSEDKSTQTIGRELKKETTSAFSQRESQPSKARMVAQVAPG
P15198    LLFQSYLDNVGVQIVRQMRSGERFLKIWSQTVEEIVSYVTVNFPNPPRRSSEDKSTQTTGRELKKETTSAFSQRESQPSKARMVAQVAPG
AFN27424  LLFQSYLDNVGVQIVRQMRSGERFLKIWSQTVEEIVSYVTVNFPNPPRRSSEDKSTQTTGRELKKETTSAFSQRESQPSKARMVAQVAPG
BBB16467  ILFQSYLDNVGVQIVRQMRSGERFLKIWSQTVEEIVSYVTVNFPNPPRRSSEDKSTQTTGRELKKETTSAFSQRESQPSKARMAAQVAPG
AAZ07892  LLFQSYLDNVGVQIVRQMRSGERFLKIWSQTIEEIVSYVTVNFPNPPRRSSEDKSTQTTGRELKKETTSAFSQRESQPSKARMVAQVASG
AAK55053  LLFQSYLDNVGVQIVRQMRSGERFLKIWSQTVEEIVSYVTVNFPNPPRRSSEDKSTQTTGRELKKETTSAFSQRESQPSKARMVAQVAPG
APD76552  LLFQSYLDNVGVQIVRQMRSGERFLKIWSQTVEEIISYVTVNFPNPPGRSSEDKSTQTTGRELKKETTSTHSQRESQPSKARMAAQSASG
Q0GBY3    LLFQSYLDNVGVQIVRQMRSGERFLKIWSQTVEEIVSYVTVNFPNPPRRSSENKSTQTTGRELKKETTSAFSQRESQPSKARMVAQVAPG
SOF05866  LLFQSYLDNVGVQIVRQMRSGERFLKIWSQTVEEIISYVTVNFPNPPGRSSEDKSTQTTGRELKKETTSTHSQRESQPSKARMAAQSASG
AFN24401  LLFQSYLDNVGVQIVRQMRSGERFLKIWSKTVEEIISYVTVNFPNPPGRSSEDKSTQTTGRELKKETTSTPPQRESQPSKARMVAQAASG
APD76597  LLFQSYLDNVGVQIVRQMRSGERFLKIWSQTVEEIISYVTVNFPNPPGRSSEDKSTQTTGRELKKETTSTHSQRESQPSKARMAAQSASG
ABF82951  LLFQSYLDNVGVQVVRPMRSLERFLKIWSQTVEEIVSYVTVNFPNPPRRSSEDKSTQTTGRELKKETTSAFSQRESQPSKARMVAQVAPG
ASU55987  LLFQSYLDNVGVQIVRQMRSGERFLKIWSQTVEEIISYVTVNFPSPPGRSSEDKSTQTTGRELKKETTSNHSQRESQPSKARMAAQSASG
AAY32477  LLFQSYLNNVGVQIVRQMRSGERFLKIWSQTVEEIVSYVTVNFPNPPRRSSEDKSTQTTGRELKKETTSAFSQRGSQPSKARMVAQVAPG
APD76602  LLFQSYLDNVGVQIVRQMRSGERFLKIWSQTVEEIISYVTVNFPNPSGRSSEDKSTQTTGRELKKETISSHSQRESQPSKVKMAAQSASG
ADU55579  LLFQSYLDNVGVQIVRQMRSGERFLKIWSQTVEEIISYVTVNFPNPPGRSSEDKSTQTTGRELKKETTSNHSQRESQPSKARMAAQSASG
ALC74030  LLFQSYLDNVGVQIVRQMRSGERFLKIWSQTVEEIISYVTVNFPSPPGRSSEDKSTQTTSRELKKETTSSHSQRESQPSKARMAAQSASG
AAK55039  LLFQSYLDNVGVQIVRQMRSGERFLKIWSQTVEEIISYVTVNFPNPPGRSSEDKSTQTTGRELKKETTSTHSQRESQPSKARMAAQSASG
AAK55015  LLFQSYLDNVGVQIVRQMRSGERFLKIWSQTVEEIISYVTVNFPNPPGRSSEDKSTQTTGKEIKKETISSHSQRESQPSKVRMAAQSASG
ASU55977  LLFQSYLDNVGVQIVRQMRSGERFLKIWSQTVEEIISYVTVNFPNPPGRSSEDKSTQTAGRELKKETTSNHSQRESQPSKARMAAQSASG
AAK55034  LLFQSYLDNVGVQIVRQMRSGERFLKIWSQTVEEIISYVTVNFPNPPGRSSEDKSTQTTGKEIKKETISSHSQRESQPSKVRMAAQSASG
AFN24256  LLLQSYLDNVGVQIVRQMRSGERFLKIWSQTVEEIISYVTVNFPNPPGSSSEDKSTQTTGRDLKKETTSPLFQRESQPSKAKMVAQVASG
AUT19348  LLFQSYLDNVGVQIVRQMRSGERFLKIWSQTVEEIISYVTVNFPNPPGRSSEDKSTQTTGREPKKETTSTPSQRESQSSKARMAAQAASG
AAK55048  LLFQSYLDNVGVQIVRQMRSGERFLKIWSQTVEEIISYVTVNFPNPSGRSSEDKSTQTTGREPKKETTSIPSQRESQSSKARMAAQAASG
AUT19448  LLFQSYLDNVGVQIVRQMRSGERFLKIWSQTVEEIISYVTVNFPNPPGRSSEDKSTQTTGREPKKETTSTPSQRESQSSKARMAAQAASG
APD76997  LLFQSYLDNVGVQIVRQMRSGERFLKIWSQTVEEIISYVTVNFPNPPGRSSEDKSTQTTGREPKKETTSTPSQRESQSSKARMAAQAASG
```

```
AUT19373  LLFQSYLDNVGVQIVRQMRSGERFLKIWSQTVEEIISYVTVNFPNPPGRSSEDKSTQTTGREPKKETTSTPSQRESQSSKARMAAQASSG
AAK55042  LLFQSYLDNVGVQIVRQMRSGERFLKIWSQTVEEIVSYVTVNFPNPPRRSSENKSTQTTGRELKKETTSAFSQRESQPSKARMVAQVAPG
QEU57682  LLFQSYLDNVGVQIVRQMRSGERFLKIWSQTVEEIISYVTVNFPNPPGRSSEDKSTQTTGREPKKETTSTPSQRESQSSKARMAAQAASG
QDZ26173  LLFQSYLDNVGVQIVRQMRSGERFLKIWSQTVEEIISYVTVNFPNPPGRSSEDKSTQTTGREPKKETTSTPSQRESQSSKARMAAQAASG
AUT19463  LLFQSYLDNVGVQIVRQMRSGERFLKIWSQTVEEIISYVTVNFPNPPGRSSEDKSTQTTSRESKKETTSTPSQRESQSSKARMAAQAASG
QDF46328  LLFQSYLDNVGVQIVRQMRSGERFLKIWSQTVEEIISYVTVNFPNPSGRSSEDKSTQTTGREPKKETTSTPSQRESQSSKARMAAQAASG
APD76752  LLFQSYLDNVGVQIVRQMRSGERFLKIWSQTVEEIVSYVTVNFPNPPGRSSEDKSTQTAGREPKKETTSNPSQRESQPSKARMAAQAASG
AAK55049  LLFQSYLDNVGVQIVRQMRSGERFLKIWSQTVEEIISYVTVNFPNSPGRSSEDKSTQTTGRESRRETTSTPSQRESQSSKAKMAAQAASG
AUT19488  LLFQSYLDNVGVQIVRQMRSGERFLKIWSQTVEEIISYVTVNFPNPPGRSSEDKSTQTTSREPKKETTSTPSQRESQSSKARMAAQAASG
AAK55004  LLFQSYLDNVGVQIVRQMRSGERFLKIWSQTVEEIISYVTVNFPNPSGRSSEDKSTQTTGREPKKETTSTPSQRESQSSRAKMAAQAASG
ARM53424  LLFQSYLDNVGVQIVRQMRSGERFLKIWSQTVEEIISYVTVNFPNPSGRSSEDKSTQTTGREPKKETTSTPSQRESQSSKARMAAQAASG
APD76857  LLFQSYLDNVGVQIVRQMRSGERFLKIWSQTVEEIISYVTVNFPNPPGRSSEDKSTQTTGREPRKETTSTPSQRESQSSKAKMAAQAASG
CUI02212  LLFQSYLDNVGVQIVRQMRSGERFLKIWSQTVEEIISYVTVNFPNPPGRSSEDKSTQTTGREPKKETTSTPSQRESQSSKARMAAQAASG
APD76852  LLFQSYLDNVGVQIVRQMRSGERFLKIWSQTVEEIISYVTVNFPNPPGRSSEDKSTQTTGREPRKETTSTPSQRESQSSKARMAAQAASG
AUT19538  LLFQSYLDNVGVQIVRQMRSGERFLKIWSQTVEEIISYVTVNFPNPPGRSSEDKSTQTVGREPKKETTSNPSQRESQPSKARMAAQAASG
AAK54999  ILFQSYLDNVGVQIVRQMRSGERFLKIWSQTVEEIISYVTVNFPNPPGRSSEDKSTQTAGREPKKGTTSTPSQRESQSSKARMAAQAASG
APD76757  LLFQSYLDNVGVQIVRQMRSGERFLKIWSQTVEEIISYVTVNFPNPPGRSSEDKSTQTAGREPKKETASNPSQRESQPSKARMAAQAASG
APD76927  LLFQSYLDNVGVQMVRQMRSGERFLKIWSQTVEEIISYVTVNFPNPPGRSSEDKSTQTTSREPKKETTSTPSQRESQSSKARMATQAASG
AUT19363  LLFQSYLDNVGVQIVRQMRSGERFLKIWSQTVEEIISYVTVNFPNLPGRSSEDKSTQTTGREPKKETTSTPSQRESQSSKARMAAQAASG
ADR67361  LLFQSYLDNVGVQIVRQMRSGERFLKIWSQTVEEIISYVTVNFPNPSGRSSEDKSTQTTGREPKKETTSTPSQRESQSSKARMAAQAASG
AUT19543  LLFQSYLDNVGVQIVRQMRSGERFLKIWSQTVEEIISYVTVNFPNPPGRPSEDKSTQTTGREPKKETTSTPSQRESQSSKARMAAQAASG
AUT19393  LLFQSYLDNVGVQIVRQMRSGERFLKIWSQTVEEIISYVTVNFPNPPGRSSEDKSTQTTGREPKKETTSTPSQRESQSSKARMAAQAASG
APD77002  LLFQSYLDNVGVQIVRQMRSGERFLKIWSQTVEEIISYVTVNFPNPPGRSSEDKSTQTTGREPKKETTSTPSQRESQSSKARMAAQAASG
AAK55038  LLFQSYLDNVGVQIVRQMRSGERFLKIWSQTVEEIISYVTVNFPNPPGRSSEDKSTQTTGREPKKETTSTPSQRESQSSKARMAAQAASG
AFN24101  LLLQSYLDNVGVQIVRQMRSGERFLKIWSQIVEEIISYVTVNFPNPPGRSSEDKSTQTTGRDLKKETTSPLSQRESQPSKARMVAQAASG
APD76687  LLFQSYLDNVGVQIVRQMRSGERFLKIWSQTVEEIISYVTVNFPNPPGRSSEDKSTQTAGREPKKGTTSTPSQRESQSSKARMAAQAASG
QEU57887  LMFQSYLDNVGVQIVRQMRSGERFLKIWSQTVEEIISYVTVNFPNPSGRSSEDKSTQTTSREPKKETTSTPSQRESQSSKARMAAQAASG
APD76932  LLFQSYLDNVGVQMVRQMRSGERFLKIWSQTVEEIISYVTVNFPNPPGRSSEDKSTQTTSREPKRETTSTPSQRESQSSKARMATQAASG
APD76872  LLFQSYLDNVGVQIVRQMRSGERFLKIWSQTVEEIISYVTVNFPNPPGRSSEDKSTQTTGREPRKETTSTPSQKESQSSKARMAAQAASG
APD76847  LLFQSYLDNVGVQIVRQMRSGERFLKIWSQTVEEIISYVTVNFPNPTGRSSEDKATQTTGREPKKETTSTPSQRESQSSKARMAAQAASG
APD76867  LLFQSYLDNVGVQIVRQMRSGERFLKIWSQTVEEIISYVTVNFPNPPGRSSEDKSTQTTGREPRKETTSTPSQRESQSSKARMAAQAASG
AAK55005  LLFQSYLDNVGVQIVRQMRSGERFLKIWSQTVEEIISYVTVNFPNPPGRSSEDKSTQTAGREPKKGTTSTPSQRESQSSRARMAAQAASG
APD76702  LLFQSYLDNVGVQIVRQMRSGERFLKIWSQTVEEIISYVTVNFPNPPGRSSEDKSTQTAGREPKKGTTSTPSQRESQSSKARMAAQAASG
QEU57512  LLFQSYLDNVGVQIVRQMRSGERFLKIWSQTVEEIISYVTVNFPNPSGRSSEDKSTQTTSREPKKETTSTPSQRESQSSKARMAAQAASG
APD76742  LLFQSYLDNVGVQIVRQMRSGERFLKIWSQTVEEIISYVTVNFPNPPGRSSEDKSTQTAGREPRKETTSNPSQRESQPSKARMAAQAASG
APD76767  LLFQSYLDNVGVQIVRQMRSGERFLKIWSQTVEEIISYVTINFPNPPGRSSEDKSTQTAGREPKKETTSNPSQRESQPSKARMAAQAASG
AUT19478  LLFQSYLDNVGVQIVRQMRSGERFLKIWSQTVEEIISYVTVNFPNPPGRSSEDKSTQTTGREPRKETTSTPSQRESQSSKVRMAAQAASG
AGQ16829  LLFQSYLDNVGVQIVRQMRSGERFLKIWSQTVEEIISYVTVNFPNPPGRSSEDKSTQTTGRESKKETTSTPSQRESQSSKARMAAQAASG
APD76937  LLFQSYLDNVGVQMVRQMRSGERFLKIWSQTVEEIISYVTVNFPNPPGRSSEDKSTQTTSREPKKETTSTPSRRESQSSKARMATQAASG
AMX81156  LLFQSYLDNVGVQMVRQMRSGERFLKIWSQTVEEIISYVTVNFPNPSGRSSEDKSTQTTGREPKKETTSAPSQREGQSSKAKMAAQAASG
QEU57537  LLFQSYLDNVGVQIVRQMRSGERFLKIWSQTVEEIISYVTVNFPNPPGRSSEDKSTQTTGRESKKETTSTTSQRESQSSKARMAAQAASG
AGQ16839  LLFQSYLDNVGVQMVRQMRSGERFLKIWSQTVEEIISYVTVNFPNPSGRSSEDKSTQTTGREPKKETTSTPSQRESQSSKAKMAAQAASG
AUT19388  LLFQSYLDNVGVQIVRQMRSGERFLKIWSQTVEEIISYVTVNFPNPPGRSSEDKSTQTTGREPKKEATSTPSQRESQSSKARMAAQAASG
CUI02217  LLFQSYLDNVGVQIVRQMRSGERFLKIWSQTVEEIISYVTVNFPNPPGRSSEDKSTQTAGREPKKGTTSTPSQRESQSSKARMAAQAASG
APD76697  LLFQSYLDNVGVQIVRQMRSGERFLKIWSQTVEEIISYVTVNFPNPPGRSSEDKSTQTAGREPKKGTTSTPSQRESQSSKARMAAQAASG
AMX81026  LLFQSYLDNIGVQMVRQMRSGERFLKIWSQTVEEIISYVTVNFPNPSGRSSEDKSTQTTGREPKKETTSTPSQRESQSSKAKMAAQAASG
AAK55001  LLFQSYLDNVGVQMVRQMRSGERFLKIWSQTVEEIISYVTVNFPNPSGRSSEDKSTQTTGREPKKETTSTPSQRESQSSKAKMAAQAASG
QEU57502  LLFQSYLDNVGVQIVRQMRSGERFLKIWSQTVEEIISYVTVNFPNPPGKSSEDKSTQTTGRESKKETTSTPSQRESQSSKARMAAQAASG
APD76977  LLFQSYLDNVGVQIVRQMRSGERFLKIWSQTVEEIISYVTVNFPNPPGRSSEDKSTQTTSREPKKETTSTPSQRESQSSKARMAAQAASG
SOF05896  LLFQSYLDNVGVQIVRQMRSGERFLKIWSQTVEEIISYVTVNFPNPSGRSSEDKSTQTTGREPKKETTSTPSQRESQSSKARMAAQAASG
AUT19573  LLFQSYLDNVGVQIVRQMRSGERFLKIWSQTVEEIISYVTVNFPNPSGRSSEDKSTQTTGREPKKETTSTPSQRESQSSKARMAAQAASG
AMX81051  ILFQSYLDNVGVQMVRQMRSGERFLKIWSQTVEEIISYVTVNFPNPSGRSSEDKSTQTTGREPKKETTSTPSQRESQSSKAKMAAQAASG
AMX80911  LLFQSYLDNVGVQMVRQMRSGERFLKIWSQTVEEIISYVTVNFPNPSGRSSEDKSTQTTSREPKKETTSTPSQRESQSSKAKMAAQAASG
APD76747  LLFQSYLDNVGVQIVRQMRSGERFLKIWSQTVEEIISYVTVNFPNPLGRSSEDKSTQTAGREPKKETTSNPSQRESQPSKARMAAQAASG
AMA67989  LLFQSYLDNVGVQIVRQMRSGERFLKIWSQTVEEIISYVMVNFPNPSGRSSEDKSTQTTGREPKKETASIPSQRESQSSKARMAAQVASG
AMX81166  LLFQSYLDNVGVQMVRQMRSGERFLKIWSQTVEEIISYVTVNFPNPSGRSSEDKSTQTTGREPKKETTSAPSQRESQSSKARMAAQAASG
APD76837  LLFQSYLDNVGVQIVRQMRSGERFLKIWSQTVEEIISYVTVNFPNPPGRSLEDKSTQTTGREPLKETTSTPSQRESQSSKAKMAAQAASG
AMA68025  LLFQSYLDNVGVQIVRQMRSGERFLKIWSQTVEEIISYVTVNFPNPSGRSSEDKSTQTTGREPKKETTSTPSQRESQSSKARMAAQAASG
APD77102  LLFQSYLDNVGIQIVRQMRSGERFLKIWSQTVEEIISYVTVNFPNPPGRSSEDKSTQTTGREPKKETTSTPSQRESQSSKARMAAQAASG
AAK55060  LLFQSYLDNIGVQIVRQMRSGERFLKIWSQTVEEIISYVTVNFPNPSGRSSEDKSTQTTGREPKKETPSTPSQRESQSSKARMAAQAASG
QKS69474  LLFQSYLDNVGVQMVRQMRSGERFLKIWSQTVEEIISYVTVNFPNPSGRPSEDKSTQTTGRELKKETTSTPSQRESQSSKARMAAQAASG

                    190       200       210       220       230       240       250       260       270
          ....|....|....|....|....|....|....|....|....|....|....|....|....|....|....|....|....|....|
P22363    PPALEWSATNEEDDLSVEAEIAHQIAESFSKKYKFPSRSSGIFLYNFEQLKMNLDDIVKEAKNVPGVTRLAHDGSKIPLRCVLGWVALAN
Q8B6J8    PPSLEWSATNGEDDLSVEAEIAHQIAESFSKKYKFPSRSSGIFLYNFEQLEMNLDDIVKEAKNVPGVTRLAHDGSKIPLRCVLGWVALAN
ADD84788  PPSLEWSATNEEDDLSVEAEIAHQIAESFSKKYKFPSRSSGIFLYNFEQLEMNLDDIVKEAKNVPGVTRLAHDGSKIPLRCVLGWVALAN
AAK54990  PPSLEWSATNEEDDLSVEAEIAHQIAESFSKKYKFPSRSSGIFLYNFEQLKMNLDDIVKEAKNVPGVTRLAHDGSKIPLRCVLGWVALAN
AAK55055  PPSLEWSATNEEDDLSVEAEIAHQIAESFSKKYKFPSRSSGIFLYNFEQLKMNLDDIVKEAKNVPGVTRLAHDGSKIPLRCVLGWVALAN
ADK90925  PPSLEWSATNGEDDLSVEAEIAHQIAESFSKKYKFPSRSSGIFLYNFEQLEMNLDDIVKEAKNVPGVTRLAHDGSKIPLRCVLGWVALAN
ACI01063  PPSLEWSATNGEDDLSVEAEIAHQIAESFSKKYKFPSRSSGIFLYNFEQLEMNLDDIVKEAKNVPGVTRLAHDGSKIPLRCVLGWVALAN
ADK90892  PPSLEWSATNGEDDLSVEAEIAHQIAESFSKKYKFPSRSSGIFLYNFEQLETNLDDIVKEAKNVPGVTRLAHDGSKIPLRCVLGWVALAN
ACL98055  PPSLEWSATNEEDDLSVEAEIAHQIAESFSKKYKFPSRSSGIFLYNFEQLEMNLDDIVKEAKNVPGVTRLAHDGSKIPLRCVLGWVALAN
```

```
ABV64427  PPSLEWSATNGEDDLSVEAEIAHQIAESFSKKYKFPSRSSGIFLYNFEQLEMNLDDIVKEAKNVPGVTRLAHDGSKIPLRCVLGWVALAN
AAZ07887  PPSLELSATNEEDDLSVEAEIAHQIAESFSKKYKFPSRSSGIFLYNFEQLKMNLDDIVKEAKNVPGVTRLAHDGSKIPLRCVLGWVALAN
CAA40929  PPALEWSATNEEDDLSVEAEIAHQIAESFSKKYKFPSRSSGIFLYNFEQLKMNLDDIVKEAKNVPGVTRLAHDGSKIPLRCVLGWVALAN
AAK54997  PPALEWSATNEEDDLSVEAEIAHQIAESFSKKYKFPSRSSGIFLYNFEQLKMNLDDIVKEAKNVPGVTRLAHDGSKIPLRCVLGWVALAN
AEK98547  PPALEWSATNEEDDLSVEAEIAHQIAESFSKKYKFPSRSSGIFLYNFEQLKMNLDDIVKEAKNVPGVTRLAHDGSKIPLRCVLGWVALAN
P15198    PPALEWSATNEEDDLSVEAEIAHQIAESFSKKYKFPSRSSGIFLYNFEQLKMNLDDIVKEAKNVPGVTRLAHDGSKIPLRCVLGWVALAN
AFN27424  PPALEWSATNEEDDLSVEAEIAHQIAESFSKKYKFPSRSSGIFLYNFEQLKMNLDDIVKEAKNVPGVTRLAHDGSKIPLRCVLGWVALAN
BBB16467  PPALEWSATNEEDDLSVEAEIAHQIAESFSKKYKFPSRSSGIFLYNFEQLKMNLDDIVKEAKNVPGVTRLAHDGSKIPLRCVLGWVALAN
AAZ07892  PPALEWSATNEEDDLSVEAEIAHQIAESFSKKYKFPSRSSGILLYNFEQLKMNLDDIVKEAKNVPGVTRLAHDGSKIPLRCVLGWVALAN
AAK55053  PPALEWSATNEEDDLSVEAEIAHQIAESFSKKYKFPSRSSGIFLYNFEQLKMNLDDIVKEAKNVPGVTRLAHDGSKIPLRCVLGWVALAN
APD76552  PPALEWTATNEEDDLSVEAEIAHQIAESFSKKYKFPSRSSGIFLYNFEQLKMNLDDIVKEAKNVPGVTRLAHDGSKIPLRCVLGWVALAN
Q0GBY3    PPALEWSATNEEDDLSVEAEIAHQIAESFSKKYKFPSRSSGIFLYNFEQLKMNLDDIVKEAKNVPGVTRLAHDGSKIPLRCVLGYVALAN
SOF05866  PPALEWTATNEEDDLSVEAEIAHQIAESFSKKYKFPSRSSGIFLYNFEQLKMNLDDIVKEAKNVPGVTRLAHDGSKIPLRCVLGWVALAN
AFN24401  PPALDWSATNEEDDLSVEAEIAHQIAESFSKKYKFPSRSSGIFLYNFEQLKMNLDDIVKEAKNVPGVTRLAHDGSKIPLRCVLGWVALAN
APD76597  PPALEWTATNEEDDLSVEAEIAHQIAESFSKKYKFPSRSSGIFLYNFEQLKMNLDDIVKEAKNVPGVTRLAHDGSKIPLRCVLGWVALAN
ABF82951  PPALEWSATNEEDDLSVEAEIAHQIAESFSKKYKFPSRSSGIFLYNFEQLKMNLDDIVKEAKNVPGVTRLAHDGSKIPLRCVLGWVALAN
ASU55987  PPALEWTATNEEDDLSVEAEIAHQIAESFSKKYKFPSRSSGIFLYNFEQLKMNLDDIVKEAKNVPGVTRLAHDGSKIPLRCVLGWVALAN
AAY32477  PPALEWSATNEEDDLSVEAEIAHQIAESFSKKYKFPSRSSGLFLYNFEQLKMNLDDIVKEAKNVPGVTRLAHDGSKIPLRCVLGWVALAN
APD76602  PPALEWTATNEEDDLSVEAEIAHQIAESFSKKYKFPSRSSGIFLYNFEQLKMNLDDIVKEAKNVPGVTRLAHDGSKIPLRCVLGWVALAN
ADU55579  PPALEWTATNEEDDLSVEAEIAHQIAESFSKKYKFPSRSSGIFLYNFEQLKMNLDDIVKEAKNVPGVTRLAHDGSKIPLRCVLGWVALAN
ALC74030  PPALEWTATNEEDDLSVEAEIAHQIAESFSKKYKFPSRSSGIFLYNFEQLKMNLDDIVKEAKNVPGVTRLAHDGSKIPLRCVLGWVALAN
AAK55039  PPALEWTATNEEDDLSVGAEIAHQIAESFSNKYKFPSRSSGIFLYNFEQLKMNLDDIVKEAKNVPGVTRLAHDGSKIPLRCVLGWVALAN
AAK55015  PPALEWTATNEEDDLSVEAEIAHQIAESFSKKYKFPSRSSGIFLYNFEQLKMNLDDIVREAKNVPGVTRLAHDGSKIPLRCVLGWVALAN
ASU55977  PPALEWTATNEEDDLSVEAEIAHQIAESFSKKYKFPSRSSGIFLYNFEQLKMNLDDIVKEAKNVPGVTRLARDGSKIPLRCVLGWVALAN
AAK55034  PPALEWTATNEEDDLSVEAEIAHQIAESFSKKYKFPSRSSGIFLYNFEQLKMNLDDIVREAKNVPGVTRLAHDGSKIPLRCVLGWVALAN
AFN24256  PPALEWSATNEEDNLSVEAEIAHQIAESFSKKYKFPSRSSGIFLYNFEQLKMNLDDIVKEAKNVPGVIRLAHDGSKIPLRCVLGWVALAN
AUT19348  PPALEWSATNEEDDLSVEAEIAHQIAESFSKKYKFPSRSSGIFLYNFEQLKMNLDDIVKEAKNVPGVTRLAHDGSKLPLRCVLGWVALAN
AAK55048  PPALEWSATNEEDDLSVEAEIAHQIAESFSKKYKFPSRSSGIFLYNFEQLKMNLDDIVKEAKNVPGVTRLAHDGSKLPLRCVLGWVALAN
AUT19448  PPALEWSATNEEDDLSVEAEIAHQIAESFSKKYKFPSRSSGIFLYNFEQLKMNLDDIVKEAKNVPGMTRLAHDGSKLPLRCVLGWVALAN
APD76997  PPALEWSATNEEDDLSVEAEIAHQIAESFSKKYKFPSRSSGIFLYNFEQLKMNLDDIVKEAKNVPGVTRLAHDGSKLPLRCVLGWVALAN
AUT19373  PPALEWSATNEEDDLSVEAEIAHQIAESFSKKYKFPSRSSGIFLYNFEQLKMNLDDIVKEAKNVPGVTRLAHDGSKLPLRCVLGWVALAN
AAK55042  PPALEWSATNEEDDLSVEAEIAHQTAESFSKKYKFPSRSSGIFLYNFEQLKMNLDDIVKEAKNVPGVTRLAHDGSKIPLRCVLGWVALAN
QEU57682  PPALEWSATNEEDDLSVEAEIAHQIAESFSKKYKFPSRSSGIFLYNFEQLKMNLDDIVKEAKNVPGVTRLAHDGSKLPLRCVLGWVALAN
QDZ26173  PPALEWSATNEEDDLSVEAEIAHQIAESFSKKYKFPSRSSGIFLYNFEQLKMNLDDIVKEAKNVPGVTRLAHDGSKLPLRCVLGWVALAN
AUT19463  PPALEWSATNEEDDLSVEAEIAHQIAESFSKKYKFPSRSSGIFLYNFEQLKMNLDDIVKEAKNVPGVTRLAHDGSKLPLRCVLGWVALAN
QDF46328  PPALEWSATNEEDDLSVEAEIAHQIAESFSKKYKFPSRSSGIFLYNFEQLKMNLDDIVKEAKNVPGVTRLAHDGSKLPLRCVLGWVALAN
APD76752  PPALEWSATNEEDDLSVEAEIAHQIAESFSKKYKFPSRSSGIFLYNFEQLKMNLDDIVKEAKNVPGVTRLAHDGSKLPLRCVLGWVALAN
AAK55049  PPALEWSATNEEDDLSVEAEIAHQIAESFSKKYKFPSRSSGIFLYNFEQLKMNLDDIVKEAKNVPGVTRLAHDGSKLPLRCVLGWVALAN
AUT19488  PPALEWSATNEEDDLSVEAEIAHQIAESFSKKYKFPSRSSGIFLYNFEQLKMNLDDIVKEAKNVPGVTRLAHDGSKLPLRCVLGWVALAN
AAK55004  PPALEWSATNEEDDLSVEAEIAHQIAESFSKKYKFPSRSSGIFLYNFEQLKMNLDDIVKEAKNVPGVTRLAHDGSKLPLRCVLGWVALAN
ARM53424  PPALEWSATNEEDDLSVEAEIAHQIAESFSKKYKFPSRSSGIFLYNFEQLKMNLDDIVKEAKNVPGVTRLAHDGSKLPLRCVLGWVALAN
APD76857  PPALEWSATNEEDDLSVEAEIAHQIAESFSKKYKFPSRSSGIFLYNFEQLKMNLDDIVKEAKNVPGVTRLAHDGSKLPLRCVLGWVALAN
CUI02212  PPALEWSTTNEEDDLSVEAEIAHQIAESFSKKYKFPSRSSGIFLYNFEQLKMNLDDIVKEAKNVPGVTRLAHDGSKLPLRCVLGWVALAN
APD76852  PPALEWSATNEEDDLSVEAEIAHQIAESFSKKYKFPSRSSGIFLYNFEQLKMNLDDIVKEAKNVPGVTRLAHDGSKLPLRCVLGWVALAN
AUT19538  PPALEWSATNEEDDLSVEAEIAHQIAESFSKKYKFPSRSSGIFLYNFEQLKMNLDDIVKEAKNVPGVTRLAHDGSKLPLRCVLGWVALAN
AAK54999  PPALEWSATNEEDDLSVEAEIAHQIAESFSKKYKFPSRSSGIFLYNFEQLKMNLDDIVKEAKNVPGVTRLAHDGSKLPLRCVLGWVALAN
APD76757  PPALEWSATNEEDDLSVEAEIAHQIAESFSKKYKFPSRSSGIFLYNFEQLKMNLDDIVKEAKNVPGVTRLAHDGSKLPLRCVLGWVALAN
APD76927  PPALEWSATNEEDDLSVEAEIAHQIAESFSKKYKFPSRSSGIFLYNFEQLKMNLDDIVKEAKNVPGVTRLAHDGSKLPLRCVLGWVALAN
AUT19363  PPALEWSATNEEDDLSVEAEIAHQIAESFSKKYKFPSRSSGIFLYNFEQLKMNLDDIVKEAKNVPGVTRLAHDGSKLPLRCVLGWVALAN
ADR67361  PPALEWSATNEEDDLSVEAEIAHQIAESFSKKYKFPSRSSGIFLYNFEQLKMNLDDIVKEAKNVPGVTRLAHDGSKLPLRCVLGWVALAN
AUT19543  PPALEWSATNEEDDLSVEAEIAHQIAESFSKKYKFPSRSSGIFLYNFEQLKMNLDDIVKEAKNVPGVTRLAHDGSKLPLRCVLGWVALAN
AUT19393  PLALEWSATNEEDDLSVEAEIAHQIAESFSKKYKFPSRSSGIFLYNFEQLKMNLDDIVKEAKNVPGVTRLAHDGSKLPLRCVLGWVALAN
APD77002  PPALEWSATNEEDDLSVEAEIAHQIAESFSKKYKFPSRSSGIFLYNFEQLKMNLDDIVKEAKNVPGVTRLAHDGSKLPLRCVLGWVALAN
AAK55038  PPALEWSATNEEDDLSVEAEIAHQIAESFSKKYKFPSRSSGIFLYNFEQLKMNLDDIVKEAKNVPGVTRLAHDGSKLPLRCVLGWVALAN
AFN24101  PPALEWSATNEEDNLSVEAEIAHQIAESFSKKYKFPSRSSGIFLYNFEQLKMNLDDIVKEAKNVPGVIRLAQDGSKIPLRCVLGWVALAN
APD76687  PPALEWSATNEEDDLSVEAEIAHQIAESFSKKYKFPSRASGIFLYNFEQLKMNLDDIVKEAKNVPGVTRLAHDGSKLPLRCVLGWVALAN
QEU57887  PPALEWSATNEEDDLSVEAEIAHQIAESFSKKYKFPSRSSGIFLYNFEQLKMNLDDIVKEAKNVPGVTRLAHDGSKLPLRCVLGWVALAN
APD76932  PPALEWSATNEEDDLSVEAEIAHQIAESFSKKYKFPSRSSGIFLYNFEQLKMNLDDIVKEAKNVPGVTRLAHDGSKLPLRCVLGWVALAN
APD76872  PPALEWSATNEEDDLSVEAEIAHQIAESFSKKYKFPSRSSGIFLYNFEQLKMNLDDIVKEAKNVPGVTRLAHDGSKLPLRCVLGWVALAN
APD76847  PPALEWSATNEEDDLSVEAEIAHQIAESFSKKYKFPSRSSGIFLYNFEQLKMNLDDIVKEAKNVPGVTRLAHDGSKLPLRCVLGWVALAN
APD76867  PPALEWSATNEEDDLSVEAEIAHQIAESFSKKYKFPSRSSGIFLYNFEQLKMNLDDIVKEAKNVPGVTRLAHDGSKLPLRCVLGWVALAN
AAK55005  PPALEWSATNEEDDLSVEAEIAHQIAESFSKKYKFPSRSSGIFLYNFEQLKMNLDDIVKEAKNVPGVTRLAHDGSKLPLRCVLGWVALAN
APD76702  PPALEWSATNEEDDLSVEAEIAHQIAESFSKKYKFPSRASGIFLYNFEQLKMNLDDIVKEAKNVPGVTRLAHDGSKLPLRCVLGWVALAN
QEU57512  PPALEWSATNEEDDLSVEAEIAHQIAESFSKKYKFPSRSSGIFLYNFEQLKMNLDDIVKEAKNVPGVTRLAHDGSKLPLRCVLGWVALAN
APD76742  PPALEWSATNEEDDLSVEAEIAHQIAESFSKKYKFPSRSSGIFLYNFEQLKMNLDDIVKEAKNVPGVTRLAHDGSKLPLRCVLGWVAVAN
APD76767  PPALEWSATNEEDDLSVEAEIAHQIAESFSKKYKFPSRSSGIFLYNFEQLKMNLDDIVKEAKNVPGVTRLAHDGSKLPLRCVLGWVALAN
AUT19478  PPALEWSATNEEDDLSVEAEIAHQIAESFSKKYKFPSRSSGIFLYNFEQLKMNLDDIVKEAKNVPGVTRLAHDGSKLPLRCVLGWVALAN
AGQ16829  PPALEWSTTNEEDDLSVEAEIAHQIAESFSKKYKFPSRSSGIFLYNFEQLKMNLDDIVKEAKNVPGVTRLAHDGSKLPLRCVLGWVALAN
APD76937  PPALEWSATNEEDDLSVEAEIAHQIAESFSKKYKFPSRSSGIFLYNFEQLKMNLDDIVKEAKNVPGVTRLAHDGSKLPLRCVLGWVALAN
AMX81156  PPALEWSATNEEDDLSVEAEIAHQIAESFSKKYKFPSRSSGIFLYNFEQLKMNLDDIVKEAKNVPGVTRLAHDGSKLPLRCVLGWVALAN
QEU57537  PPALEWSTTNEEDDLSVEAEIAHQIAESFSKKYKFPSRSSGIFLYNFEQLKMNLDDIVKEAKNVPGVTRLAHDGSKLPLRCVLGWVALAN
AGQ16839  PPALEWSATNEEDDLSVEAEIAHQIAESFSKKYKFPSRSSGIFLYNFEQLKMNLDDIVKEAKNVPGVTRLAHDGSKLPLRCVLGWVALAN
AUT19388  PPALEWSATNEEDNLSVEAEIAHQIAESFSKKYKFPSRSSGIFLYNFEQLKMNLDDIVKEAKNVPGVTRLAHDGSKLPLRCVLGWVALAN
```

```
CUI02217  PPALEWSATNEEDDLSVEAEIAHQIAESFSKKYKFPSRSSGIFLYNFEQLKMNLDDIVKEAKNVPGVTRLAHDGSKLPLRCVLGWVALAN
APD76697  PPALEWSATNEEDDLSVEAEIAHQIAESFSKKYKFPSRASGIFLYNFEQLKMNLDDIVKEAKNVPGVTRLAHDGSRLPLRCVLGWVALAN
AMX81026  PPALEWSATNEEDDLSVEAEIAHQIAESFSKKYKFPSRSSGIFLYNFEQLKMNLDDIVKEAKNVPGVTRLAHDGSKLPLRCVLGWVALAN
AAK55001  PPALEWSATNEEDDLSVEAEIAHQIAESFSKKYKFPSRSSGIFLYNFEQLKMNLDDIVKEAKNVPGVTRLAHDGSKLPLRCVLGWVALAN
QEU57502  PPALEWSTTNEEDDLSVEAEIAHQIAESFSKKYKFPSRSSGIFLYNFEQLKMNLDDIVKEAKNVPGVTRLAHDGSKLPLRCVLGWVALAN
APD76977  PPALEWSATNEEDDLSVEAEIAHQIAESFSKKYKFPSRSSGIFLYNFEQLKMNLDDIVNEAKNVPGVTRLAHDGSKLPLRCVLGWVALAN
SOF05896  PPALEWSATNEEDDLSVEAEIAHQIAESFSKKYKFPSRSSGIFLYNFEQLKMNLDDIVKEAKNVPGVTRLAHDGSKLPLRCVLGWVALAN
AUT19573  PPALEWSATNEEDDLSVEAEIAHQIAESFSKKYKFPSRSSGIFLYNFEQLKMNLDDIVKEAKNVPGVTRLAHDGSKLPLRCVLGWVALAN
AMX81051  PPALEWSATNEEDDLSVEAEIAHQIAESFSKKYKFPSRSSGIFLYNFEQLKMNLDDIVKEAKNVPGVTRLAHDGSKLPLRCVLGWVALAN
AMX80911  PPALEWSATNEEDDLSVEAEIAHQIAESFSKKYKFPSRSSGIFLYNFEQLKMNLDDIVKEAKNVPGVTRLAHDGSKLPLRCVLGWVALAN
APD76747  PPALEWSATNEEDDLSVEAEIAHQIAESFSKKYKFPSRSSGIFLYNFEQLKMNLDDIVKEAKNVPGVTRLAHDGSKLPLRCVLGWVALAN
AMA67989  PPALEWSATNEEDDLSVEAEIAHQIAESFSKKYKFPSRSSGIFLYNFEQLKMNLDDIVKEAKNVPGVTRLAHDGSKIPLRCVLGWVALAN
AMX81166  PPALEWSATNEEDDLSVEAEIAHQIAESFSKKYKFPSRSSGIFLYNFEQLKMNLDDIVKEAKNVPGVTRLAHDGSKLPLRCVLGWVALAN
APD76837  PPALEWSATNEEDDLSVEAEIAHQIAESFSKKYKFPSRSSGIFLYNFEQLKMNLDDIVKEAKNVPGVTRLAHDGSKLPLRCVLGWVALAN
AMA68025  PPALEWSATNDEDDLSVEAEIAHQIAESFSKKYKFPSRSSGIFLYNFEQLKMNLDDIVKEAKNVPGVTRLAHDGSKLPLRCVLGWVALAN
APD77102  PPALEWSATNEEDDLSVEAEIAHQIAESFSKKYKFPSRSSGIFLYNFEQLKMNLDDIVKEAKNVPGVTRLAHDGSKLPLRCVLGWVALAN
AAK55060  PPALEWSATNEEDDLSVEAEIAHQIAESFSKKYKFPSRSSGIFLYNFEQLKMNLDDIVKEAKNVPGVTRLAHDGSKLPLRCVLGWVALAN
QKS69474  PPALEWSATNEEDDLSVEAEIAHQIAESFSKKYKFPSRSSGIFLYNFEQLKMNLDDIVKEAKNVPGVTRLAHDGSKLPLRCVLGWVALAN

                  280       290
          ....|....|....|....|....|..
P22363    SKKFQLLVEADKLSKIMQDDLNRYTSC
Q8B6J8    SKKFQLLVEADKLSKIMQDDLDRYTSC
ADD84788  SKKFQLLVEADKLSKIMQDDLDRYKSC
AAK54990  SKKFQLLVEADKLSKIMQDDLDRYTSC
AAK55055  SKKFQLLVEADKLSKIMQDDLDRYTSC
ADK90925  SKKFQLLVEADKLSKIMQDDLDRYTSC
ACI01063  SKKFQLLVEADKLSKIMQDDLDRYTSC
ADK90892  SKKFQLLVEADKLSKIMQDDLDRYTSC
ACL98055  SKKFQLIVEADKLSKIMQDDLDRYTSC
ABV64427  SKKFQLLVEPDKLNKIMQDDLNRYTSC
AAZ07887  SKKFQLIVEADKLSKIMQDDLDRYTSC
CAA40929  SKKFQLLVEADKLSKIMQDDLNRYTSC
AAK54997  SKKFQLLVEADKLSKIMQDDLNRYTSC
AEK98547  SKKFQLLVEADKLSKIMQDDLNRYTSC
P15198    SKKFQLLVEADKLSKIMQDDLNRYTSC
AFN27424  SKKFQLLVEADKLSKIMQDDLNRYTSC
BBB16467  SKKFQLLVEADKLSKIMQDDLNRYTSC
AAZ07892  SKKFQLLVEADKLSKIMQDDLNRYTSC
AAK55053  SKKFQLLVEADKLSKIMQDDLNRYTSC
APD76552  SKKFQLLVEADKLSKIMQDDLNRYTSA
Q0GBY3    SKKFQLLVEADKLSKIMQDDLNRYTSC
SOF05866  SKKFQLLVEADKLSKIMQDDLNRYTSV
AFN24401  SKKFQLLVEADKLGKIMQDDLNRYTSC
APD76597  SKKFQLLVEADKLSKIMQDDLNRYTSA
ABF82951  SKKFQLLVEADKLSKIMQDDLNRYTSC
ASU55987  SKKFQLLVEADKLSKIMQDDLNRYTSV
AAY32477  SKKFQLLVEADKLSKIMQDDLNRYTSC
APD76602  SKKFQLLVEADKLSKIMQDDLNRYTSC
ADU55579  SKKFQLLVEADKLSKIMQDDLNRYTS
ALC74030  SKKFQLLVEADKLSKIMQDDLNRYTSV
AAK55039  SKKFQLLVEADKLSKIMQDDLNRYTSA
AAK55015  SKKFQLLVEADKLSKIMQDDLNRYTSC
ASU55977  SKKFQLLVEADRLGKIMQDDLNRYTSC
AAK55034  SKKFQLLVEADKLSKIMQDDLNRYTSC
AFN24256  SKKFQLLVEADKLSKIMQDDLNRYTSC
AUT19348  SKKFQLLVEADKLSKIMQDDLNRYASC
AAK55048  SKKFQLLVEADKLNKIMQDDLNRYASC
AUT19448  SKKFQLLVEADKLSKIMQDDLNRYASC
APD76997  SKKFQLLVEADKLNKIMQDDLNRYASC
AUT19373  SKKFQLLVEADKLSKIMQDDLNRYASC
AAK55042  SKKFQLLVEADKLSKIMQDDLNRYTSC
QEU57682  SKKFQLLVEADKLSKIMQDDLNRYASC
QDZ26173  SKKFQLLVEADKLSKIMQDDLNRYASC
AUT19463  SKKFQLLVEADKLSKIMQDDLNRYTSC
QDF46328  SKKFQLLVEADKLNKIMQDDLNRYASC
APD76752  SKKFQLLVEADKLSKIMQDDLNRYASC
AAK55049  SKKFQLLVEADKLSKIMQDDLNRYTSC
AUT19488  SKKFQLLVEADKLSKIMQDDLNRYTSC
AAK55004  SKKFQLLVEADKLSKIMQDDLNRYTSC
ARM53424  SKKFQLLVEADKLSKIMQDDLNRYASC
APD76857  SKKFQLLVEADKLSKIMQDDLNRYASC
CUI02212  SKKFQLLVEADKLSKIMQDDLNRYASC
```

```
APD76852  SKKFQLLVEADKLSKIMQDDLNRYASC
AUT19538  SKKFQLLVEADKLSKIMQDDLNRYASC
AAK54999  SKKFQLLVEADKLSKIMQDDLNRYTSC
APD76757  SKKFQLLVEADKLSKIMQDDLNRYASC
APD76927  SKKFQLLVEADKLSKIMQDDLNRYASC
AUT19363  SKKFQLLVEADKLSKIMQDDLNRYASC
ADR67361  SKKFQLLVEADKLNKIMQDDLNRYASC
AUT19543  SKKFQLLVEADKLSRIMQDDLNRYASC
AUT19393  SKKFQLLVEADKLSKIMQDDLNRYASC
APD77002  SKKFQLLVEADKLNKIMQDDLNRYASC
AAK55038  SKKFQLLVEADKLSKIMQDDLNRYASC
AFN24101  SKKFQLLVEADKLSKIMQDDLNRYTSC
APD76687  SKKFQLLVEADKLSKIMQDDLNRYTSC
QEU57887  SKKFQLLVEADKLSKIMQDDLNRYASC
APD76932  SKKFQLLVEADKLSKIMQDDLNRYASC
APD76872  SKKFQLLVEADKLSKIMQDDLNRYASC
APD76847  SKKFRLLVEADKLSKIMQDDLNRYASC
APD76867  SKKFQLLVEADKLSKIMQDDLNRYASC
AAK55005  SKKFQLLVEADKLSKIMQDDLNRYTSC
APD76702  SKKFQLLVEADKLSKIMQDDLNRYTSC
QEU57512  SKKFQLLVEADKLSKIMQDDLNRYASC
APD76742  SKKFQLLVEADKLSKIMQDDLNRYASC
APD76767  SKKFQLLVEADKLSKIMQDDLNRYASC
AUT19478  SKKFQLLVEADKLSKIMQDDLNRYASC
AGQ16829  SKKFQLLVEADKLSKIMQDDLNRYASC
APD76937  SKKFQLLVEADKLSKIMQDDLNRYASC
AMX81156  SKKFQLLVEADKLNKIMQDDLNRYTSC
QEU57537  SKKFQLLVEADKLSKIMQDDLNRYASC
AGQ16839  SKKFQLLVEADKLNKIMQDDLNRYTSC
AUT19388  SKKFQLLVEADKLSKIMQDDLNRYASC
CUI02217  SKKFQLLVEADKLSKIMQDDLNRYTSC
APD76697  SKKFQLLVEADKLSKIMQDDLNRYTSC
AMX81026  SKKFQLLVEADKLNKIMQDDLNRYTSC
AAK55001  SKKFQLLVEADKLNKIMQDDLNRYTSC
QEU57502  SKKFQLLVEADKLSKIMQDDLDRYASC
APD76977  SKKFQLLVEADKLSKIMQDDLNRYASC
SOF05896  SKKFQLLVEADKLNKIMQDDLNRYASC
AUT19573  SKKFQLLVEADKLNKIMQDDLNRYTSC
AMX81051  SKKFQLLVEADKLNKIMQDDLNRYASC
AMX80911  SKKFQLLVEADKLNKIMQDDLNRYTSC
APD76747  SKKFQLLVEADKLSKIMQDDLNRYASC
AMA67989  SKKFQLLVEADKLNKIMQDDLNRYASC
AMX81166  SKKFQLLVEADKLNKIMQDDLNRYTSC
APD76837  SKKFQLLVEADKLSKIMQDDLNRYASC
AMA68025  SKKFQLLVEADKLNKIMQDDLNRYTSC
APD77102  SKKFQLLVEADKLSKIMQDDLNRYASC
AAK55060  SKKFQLLVEADKLNKVMQDDLNRYESC
QKS69474  SKKFQLLVEADKLNKIMQDDLNRYTSC
```

Aligned sequences of rabies virus P proteins. CVS11 sequence (P22363) and HEP-Flury (Q8B6J8) sequences are top and second from the top, respectively. Residues identical in 50% or more sequences marked with gray background.

Supplementary Figure S3.

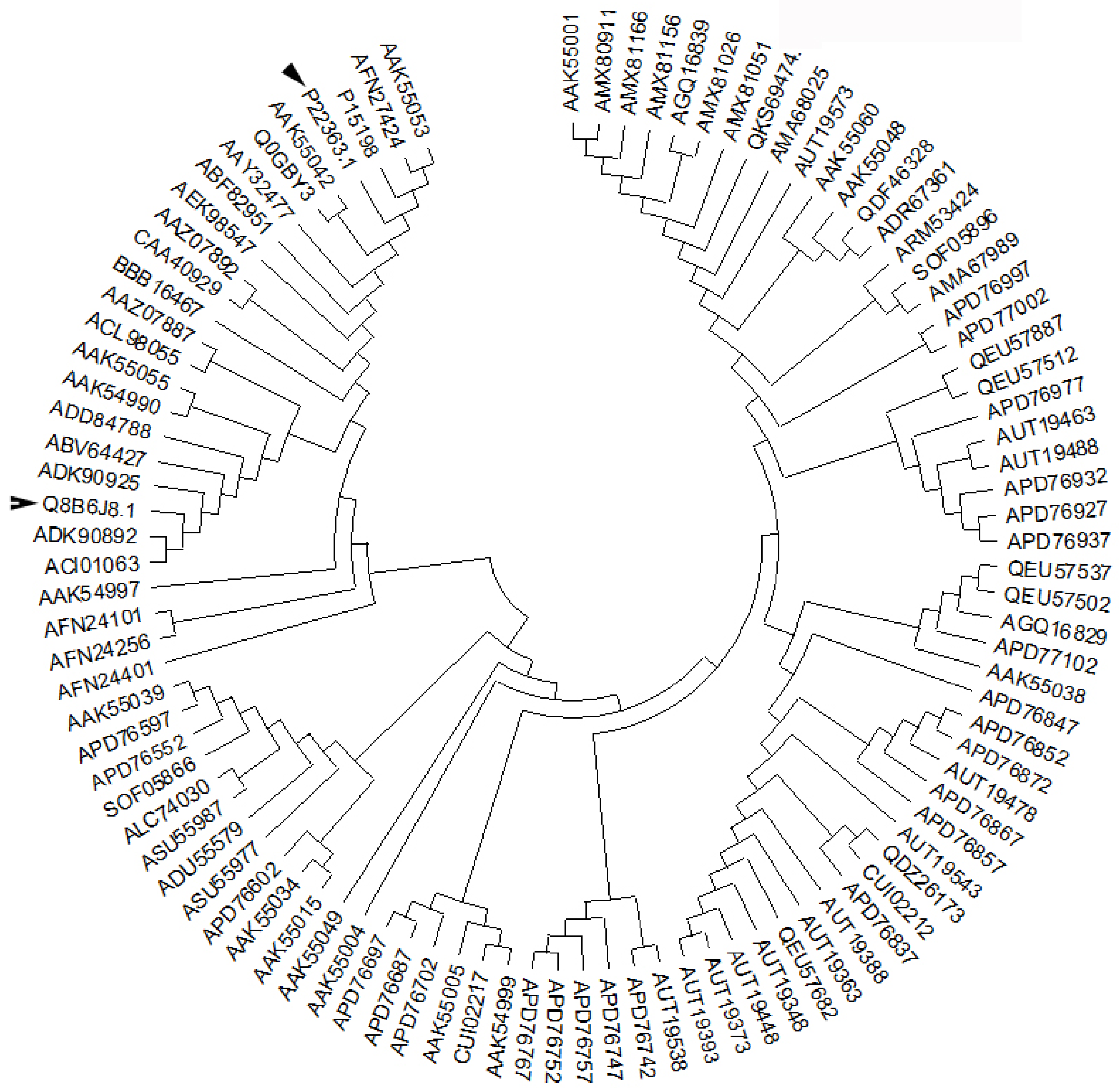

Phylogenetic tree of the P proteins presented in Supplementary fig. 2. Position of CVS-11 (P22363) and HEP-Flury (Q8B6J8) marked with arrows.
